# Within-host *Mycobacterium tuberculosis* diversity and its utility for inferences of transmission

**DOI:** 10.1099/mgen.0.000217

**Published:** 2018-10-11

**Authors:** Michael A. Martin, Robyn S. Lee, Lauren A. Cowley, Jennifer L. Gardy, William P. Hanage

**Affiliations:** ^1^​ Center for Communicable Disease Dynamics, Department of Epidemiology, Harvard T. H. Chan School of Public Health, Boston, MA, USA; ^2^​ Department of Epidemiology, Harvard University, Boston, MA 02115, USA; ^3^​ School of Population and Public Health, University of British Columbia, Vancouver, Canada; ^4^​ British Columbia Centre for Disease Control, Vancouver, Canada

**Keywords:** *Mycobacterium tuberculosis*, genomic epidemiology, transmission, whole genome sequencing, within-host diversity

## Abstract

Whole genome sequencing in conjunction with traditional epidemiology has been used to reconstruct transmission networks of *Mycobacterium tuberculosis* during outbreaks. Given its low mutation rate, genetic diversity within *M. tuberculosis* outbreaks can be extremely limited – making it difficult to determine precisely who transmitted to whom. In addition to consensus SNPs (cSNPs), examining heterogeneous alleles (hSNPs) has been proposed to improve resolution. However, few studies have examined the potential biases in detecting these hSNPs. Here, we analysed genome sequence data from 25 specimens from British Columbia, Canada. Specimens were sequenced to a depth of 112–296×. We observed biases in read depth, base quality, strand distribution and read placement where possible hSNPs were initially identified, so we applied conservative filters to reduce false positives. Overall, there was phylogenetic concordance between the observed 2542 cSNP and 63 hSNP loci. Furthermore, we identified hSNPs shared exclusively by epidemiologically linked patients, supporting their use in transmission inferences. We conclude that hSNPs may add resolution to transmission networks, particularly where the overall genetic diversity is low.

## Data Summary

1. Raw *M. tuberculosis* read files have been deposited in ﻿National Center for Biotechnology Information’s Sequence Read Archive (NCBI SRA) under accession number PRJNA413593 (Table S1, available in the online version of this article).

2. *M. tuberculosis* strain H37Rv is available from GenBank; accession number NC_000962.3.

3. *M. tuberculosis* strain CCDC5079 is available from GenBank; accession number CP001641.1.

4. Source code for processing of raw reads and variant calling is available from GitHub; https://github.com/c2-d2/within-host-diversity.

**Impact Statement**

Genomic analysis in outbreaks of pathogens such as *Mycobacterium tuberculosis* has allowed for more accurate estimation of transmission networks, aiding in control and response efforts. However, in instances when genetic diversity is low, analyses that overlook the variation within single hosts may be unable to resolve transmission. In this study, we investigate the application of such heterogeneous alleles (hSNPs) for inferring transmission. We identify critical sources of bias that need to be accounted for in the bioinformatics analysis when identifying these variants. In terms of transmission, many hSNPs identified were consistent with the consensus SNP-based approach and epidemiological data. hSNPs also provided genomic support for transmission between an epi-linked pair that would have been missed using consensus SNPs alone. Incorporating the analysis of hSNPs in future outbreaks may be important to help inform inferences of transmission.

## Introduction

*Mycobacterium tuberculosis* is the leading communicable cause of mortality worldwide, causing 1.7 million deaths in 2016 [1]. Whole genome sequencing (WGS) has been used to estimate the transmission network of *M. tuberculosis* outbreaks, with identical or highly similar genetic sequences providing support for direct transmission between patients [2–6].

However, *M. tuberculosis* is among the most homogeneous of bacterial species. Due to the low diversity of *M. tuberculosis* genomes, cases which are not epidemiologically linked can therefore have highly similar consensus sequences [7, 8]. Within-host genetic diversity of *M. tuberculosis* has previously been described [9–12] and may be due to within-host evolution or co-infection. The analysis of this diversity, i.e. making use of polymorphisms that arise during infection that may be transmitted through non-stringent bottlenecks, has been proposed to increase resolution of transmission. Modelling studies suggest that overlooking this diversity can lead to erroneous transmission inferences using genomic data alone [13]. A number of tools have been proposed to incorporate within-host diversity into transmission estimates [14–18], but their application to real world data remains limited. Furthermore, methods to reliably identify within-host diversity from WGS data are not well established.

Here we present the analysis of WGS data from *M. tuberculosis* specimens with an emphasis on the identification of consensus SNPs (cSNPs) and heterogeneous alleles (hSNPs) indicative of within-host variation. While we highlight important sources of bioinformatics biases in the identification of hSNPs, we show that hSNPs may indeed provide additional resolution beyond the cSNP-based approach for inferences of transmission.

## Theory and implementation

### Data collection

Samples were cultured at the British Columbia Centre for Disease Control Public Health Laboratory and clean sweeps of Lowenstein-Jensen (LJ) slants were sequenced at the BC Genome Sciences Centre using the Illumina HiSeq platform (Supplementary Methods) [2]. Epidemiological data (Table S1) were collected by local public health units as part of routine investigations and provided in non-nominal form. Included specimens were chosen from a larger study [19] to represent two clusters of cases defined by classical genotyping (Supplementary Methods). One of these was composed exclusively of locally transmitted cases, while the other included locally transmitted cases, epidemiologically unlinked cases arising from reactivation of tuberculosis infection acquired outside Canada, and serial specimens from a single patient.

### Genomic investigation and definition of hSNPs

**Fig. 1.**
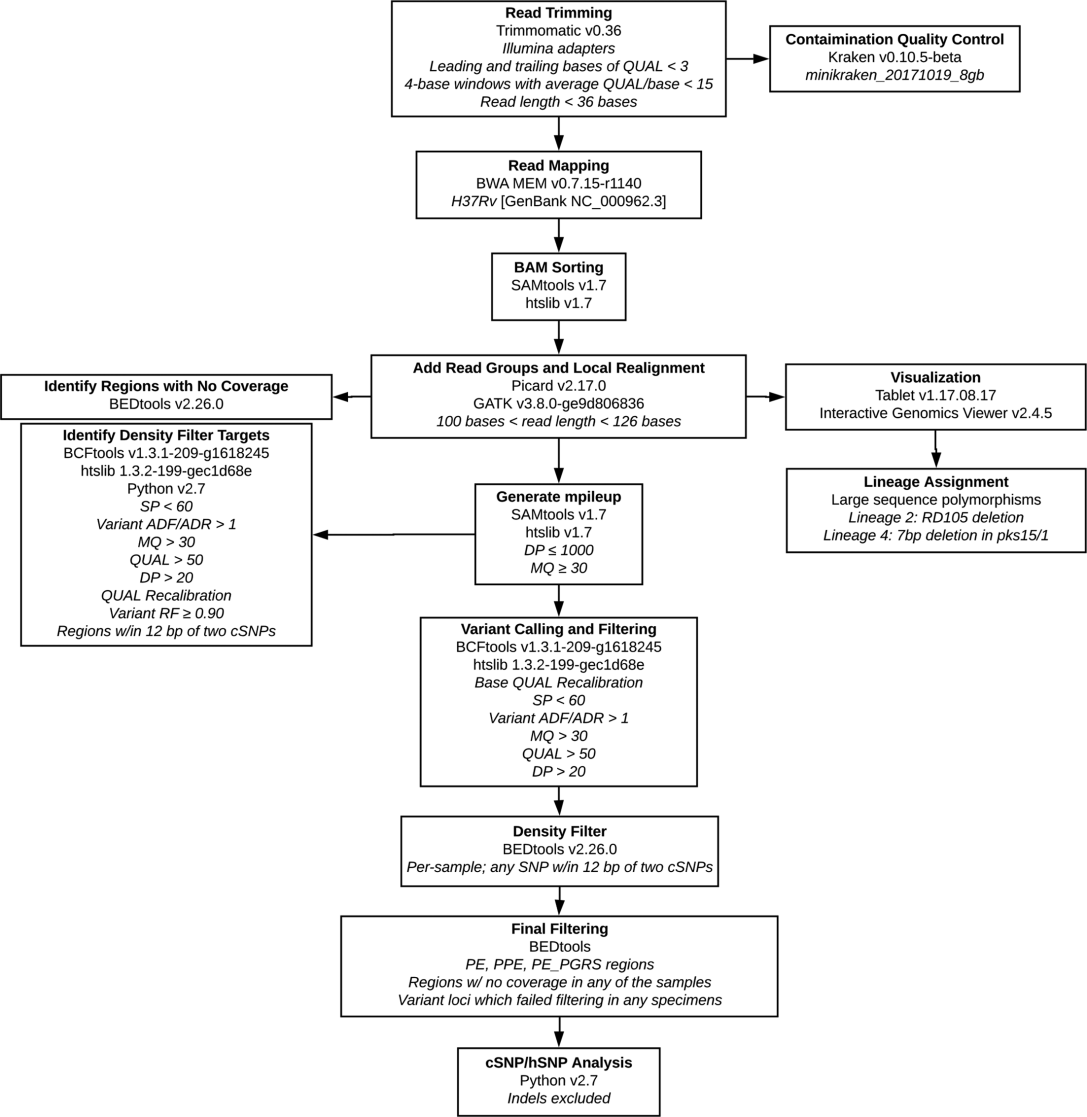
Schematic description of our final analysis pipeline (Supplementary Methods). ADF, high-quality allelic depth on the forward strand; ADR, high-quality allelic depth on the reverse strand; BAM, binary alignment map; bp: base pair; DP, high-quality read depth; Indels, small insertions and deletions; MQ, Phred-scaled average mapping quality; QUAL, Phred-scaled base quality score; SAM, sequence alignment map; SP, Phred-scaled strand bias *P*-value; PE, proline-glutamic acid; PPE, proline-proline-glutamic acid, PE_PGRS, proline-glutamic acid_polymorphic guanine-cytosine-rich sequence; RF, read frequency, high-quality variant reads/total high-quality reads.

Reads were trimmed with Trimmomatic v0.36 and aligned to the H37Rv reference genome (GenBank accession number: NC_000962.3) [20, 21] using BWA MEM v0.7.15 [22]. Local realignment was conducted using GATK v3.8.0 [23]. Identification of large sequence polymorphisms was used to assign lineage [24]. Variant calling and filtering was conducted using SAMtools v1.7 and BCFtools v1.7 [25]. Proline-glutamic acid (PE), proline-proline-glutamic acid (PPE) and proline-glutamic acid_polymorphic guanine-cytosine-rich sequence (PE_PGRS) (PE/PPE) genes were excluded, as the disproportionate number of variants in these regions was suggestive of mapping error (Fig. S1). Our complete analysis pipeline is shown in Fig. 1 and is described in the Supplementary Methods.

SNPs were considered consensus (cSNPs) if at least 90 % of the reads supported the variant allele and hSNPs if greater than 10 % and up to 90 % of the reads supported the variant allele. SNPs with 10 % or less of the reads supporting the variant allele were called as reference. Informative SNPs were defined as variants present in at least one but not all of the specimens. The possibility of co-infection with another lineage of *M. tuberculosis* was investigated by checking whether any hSNPs were present at lineage-defining alleles [26].

### Phylogenetic analysis

Concatenated SNP alignments were used as input for phylogenetic trees. hSNP alignments were generated using IUPAC ambiguity codes [27]. IQ-TREE v1.6.1 was used to infer maximum-likelihood (ML) phylogenies with ultrafast bootstrap support values as well as 100 random phylogenies [28–30]. Distance between trees was calculated using the Kendall–Colijn (KC) metric (lambda=0) [31, 32]. We also compared cSNP and hSNP phylogenies using the approximately unbiased (AU) test [33] in CONSEL v0.20 [34]. Further details are provided in the Supplementary Methods.

### Alignment-induced bias in observed hSNPs

All specimens were confirmed to be *M. tuberculosis* complex and sequenced to at least 100 average depth [median (sd), range: 178× (31.15), 112–296] (Table S2). Following initial filtering criteria [all reads with mapping quality (MQ) >30, average MQ>30, base quality (QUAL) >50, high-quality read depth (DP) >20], we identified 2945 SNP loci, where at least one specimen had a cSNP or hSNP identified (2812 loci were identified where at least one specimen had a cSNP and 167 loci were identified where at least one specimen had an hSNP; these are not mututally exclusive as cSNPs in one specimen could be hSNPs in another). After removing SNPs in PE/PPE regions, 2654 SNP loci, where at least one specimen had a cSNP or hSNP identified (2573 cSNP and 94 hSNP loci), remained. A narrower distribution of Phred-scaled stand bias (SP) scores was present amongst cSNPs as compared to hSNPs [mean (sd): 0.18 (1.28) versus 32.08 (30.74), respectively, Fig. S2]. We therefore additionally filtered for only variants with SP<60 (maximum observed cSNP SP: 58) and required >1 variant read on both strands.

Manual inspection of the alignments revealed that many positions initially called as hSNPs were probably due to alignment error, where the alternative allele was almost exclusively on reads that had been soft-clipped during mapping (Fig. S3, Table S3). Consequently, we removed all reads with less than 100 aligned base pairs (representing 3.5 % of reads with MQ >30 in all specimens) from our analysis. Seventeen loci had differential SNP calls following this filtering step; five loci which were erroneously called as hSNPs previously were subsequently called as reference and six loci which failed the filtering protocol before removing clipped reads were included following this additional step.

The final dataset included 2598 SNP loci, where at least one specimen had a cSNP or hSNP identified (2542 loci were identified where at least one specimen had a cSNP and 63 loci were identified where at least one specimen had an hSNP). cSNP distances ranged from 0 to 1237 (Table S4). hSNP bases were shared between one and 25 specimens (Fig. S4, Table S5). Specimens belonged to lineage 4 (Euro-American, *n*=5) or lineage 2 (East-Asian, *n*=20) (Table S1). There was no evidence of co-infection by multiple lineages of *M. tuberculosis.* Lineage 4 specimens were 672–675 cSNPs from H37Rv and lineage 2 specimens were 1196–1237 cSNPs from H37Rv. The number of observed hSNPs per specimen was correlated with the distance from H37Rv (*R*^2^=0.34); this suggests some inferred hSNPs may in fact be generated by alignment errors, resulting from structural polymorphisms present in our specimens which are not in the reference genome. However, mapping the lineage 2 genomes to a lineage 2 reference did not significantly impact phylogenetic analysis or transmission inferences (Supplementary Results, Tables S6 and S7, Figs S5 and S6), consistent with a previous cSNP-based study [35].

A comparison of key quality metrics after filtering is shown in Table 1 and Fig. S7. On average, read depth was higher at observed hSNPs. Strand bias, base quality bias, mapping quality bias and read position bias were all more significant amongst hSNPs. Mapping quality was similar between cSNPs and hSNPs. Given the limited number of hSNPs shared across all specimens (indicative of alignment-induced false positives) (*n*=6) compared to our initial analysis (*n*=25), we believe our filtering protocol has probably removed the majority of false positive hSNPs. Statistical tests comparing cSNP and hSNP metrics were not conducted due to the correlated nature of these data.

**Table 1.** Quality metrics comparing consensus and heterogeneous SNPs after the final filtering protocol DP, high-quality read depth; QUAL, recalibrated base quality score; MQ, average mapping quality; SP, Phred-scaled strand bias *P*-value; BQB, Mann–Whitney U test of base quality bias; MQB, Mann–Whitney U test of mapping quality bias; RPB, Mann–Whitney U test of read position bias.

	cSNP		hSNP	
	Mean	sd	Mean	sd
DP	161.84	41.34	232.84	88.17
QUAL	228.00	0.36	209.13	34.66
MQ	59.71	1.95	59.42	1.57
SP	0.16	1.02	10.44	11.10
BQB*	0.98	0.09	0.68	0.30
MQB*	1.00	0.03	0.91	0.22
RPB*	0.98	0.10	0.28	0.10

*As BQB, MQB and RPB are only defined at positions with reference and variant reads, we assume an RPB value of 1.0 for SNPs with 100 % variant reads

Given the removal of reads marked as PCR duplicates in published analyses of *M. tuberculosis* sequencing data [35], we compared whether the inclusion of this step influenced our results. No significant differences in the number of cSNPs or hSNPs was observed and epidemiological inferences remained the same (Supplementary Results, Fig. S8).

### SNPs correlate with epidemiological data

**Fig. 2.**
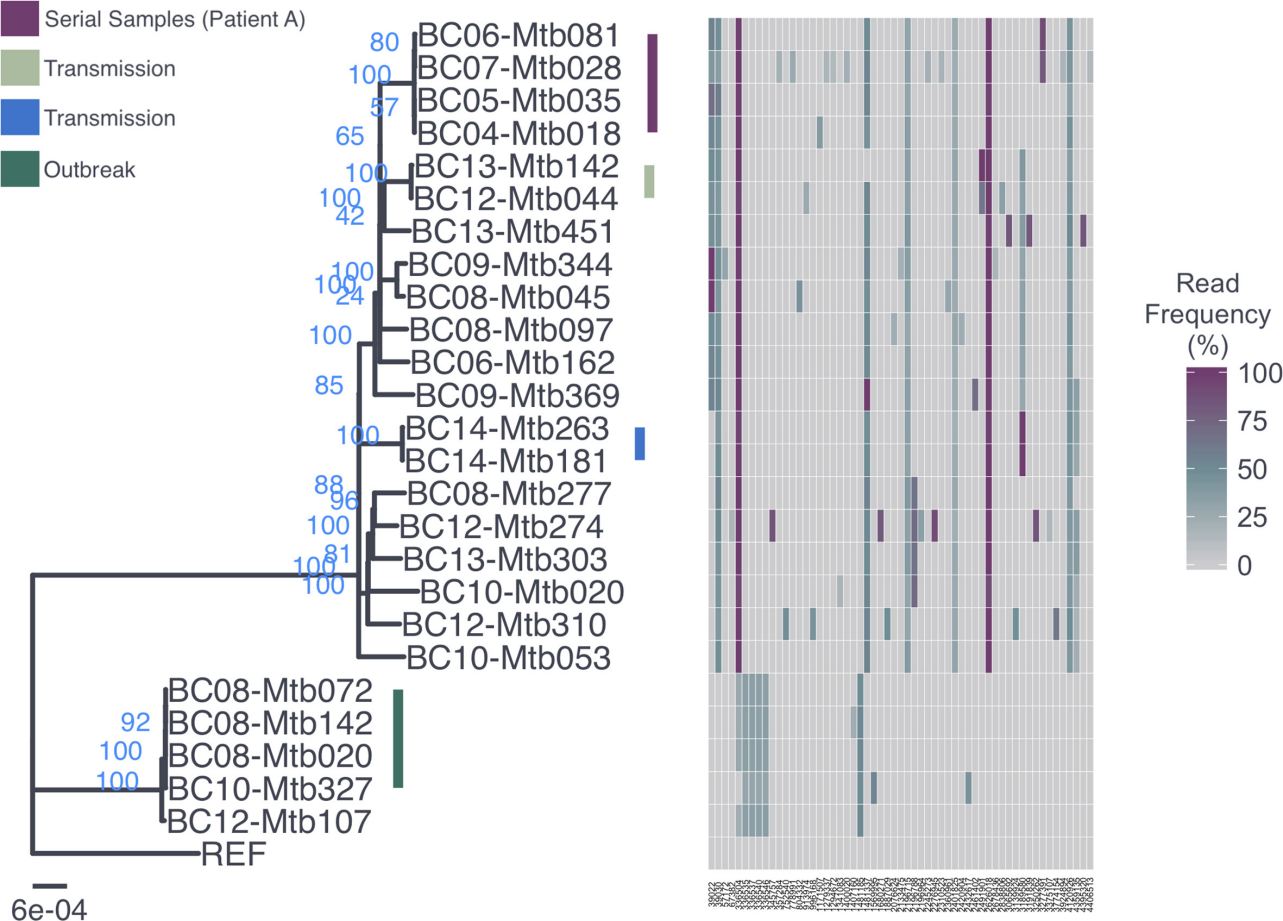
Maximum-likelihood (ML) phylogeny generated using an alignment of 2542 cSNPs, rooted on the H37Rv reference (REF). TVMe+ASC was identified as the best-fit model based on the Bayesian information criterion. Ultrafast bootstrap support values are annotated in blue (support values >95 % indicate high confidence) and epidemiological data provided by the BCCDC (British Columbia Centre for Disease Control) are indicated in the top left. Branches without annotation are not epidemiologically linked to other cases in the dataset. Each column at the right represents an informative hSNP as compared to H37Rv, ordered by position in the genome and coloured by supporting variant read frequency percentage. The scale bar corresponds to the number of subsitutions per site.

Epidemiological data suggested three separate transmission chains (*n*=2, 2 and 4) (Table S1). This correlated with the genetic data (Fig. 2, Table S4) as specimens from cases within presumed transmission chains were 0–2 cSNPs apart and were >10 cSNPs from those without epidemiological links. Specimens from epidemiologically linked cases were also phylogenetically clustered with high bootstrap *P*-values (Fig. 2).

Amongst transmission pairs (described in [36]), one had identical hSNPs (BC14-Mtb181 and BC14-Mtb263, cSNP distance: 1), while the other (BC12-Mtb044 and BC13-Mtb143, cSNP distance: 2) had an hSNP which was present in the 2012 specimen and present as a cSNP in the 2013 specimen, and three hSNPs present in the 2012 specimen were not observed in 2013. This may indicate a bottleneck during transmission, which would be consistent with the small infectious dose of *M. tuberculosis* [37].

Among the outbreak cluster involving four cases with pairwise cSNP distances between 0 and 1, there was a nearly identical pattern of hSNPs. The same hSNPs were also observed in the fifth lineage 4 specimen (BC12-Mtb107), which was 14–15 cSNPs from the outbreak cluster and not identified as being epidemiologically linked. This suggests that the observed hSNPs may be due to real underlying structural variation that is present within our specimens (and therefore epidemiologically relevant) but is not found in the reference, thereby resulting in alignment errors.

Our dataset also included four serial specimens collected between 2004 and 2007 (Table S1) from a single patient (‘Patient A’) diagnosed with multi-drug resistant (MDR) *M. tuberculosis* (Supplementary Results). These specimens differed from one another by a maximum of two cSNPs and 11 hSNPs. There was one hSNP present in the 2004 specimen that was called as reference in the remaining specimens. Two novel cSNPs, unique to these specimens, were observed in the 2006 specimens. One of these cSNPs was called as an hSNP in the 2007 specimen (82 % variant read frequency); as this would have been missed by standard consensus-based SNP calling, this illustrates that hSNPs can provide additional information when determining genetic distance. Nine additional hSNPs were present in the 2007 specimen. While these may be due to within-host evolution, they may more likely be due to sampling; the bacteria in the previous sputum samples might not have been representative of the full range of diversity generated within the lung, a limitation of all genomic analysis of *M. tuberculosis* [38]. Alternatively, while there was no evidence of co-infection with a different *M. tuberculosis* lineage, co-infection with another closely related lineage 2 strain from outside our collection or laboratory cross-contamination [39] are also possible explanations. Otherwise, observed hSNPs and cSNPs were the same for all four specimens (Supplementary Results, Table S8, Fig. S9).

**Fig. 3.**
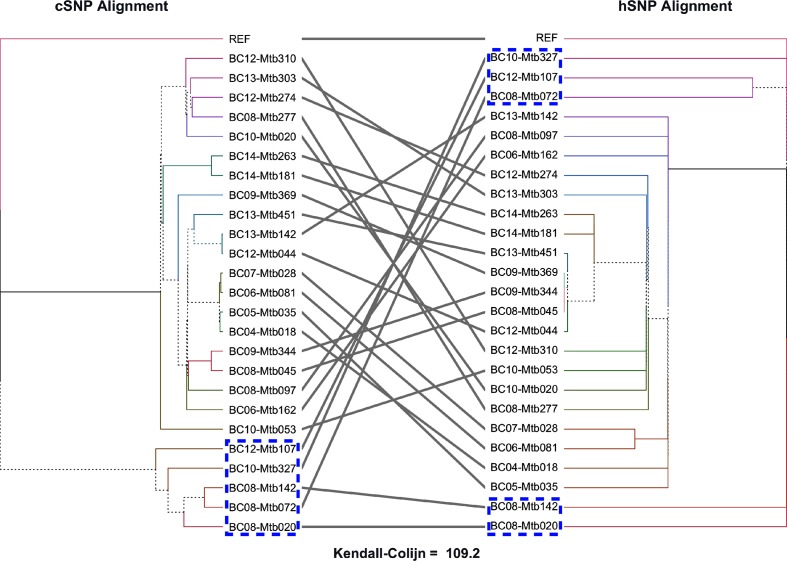
Tanglegram comparing the topology of a cSNP-defined ML phylogeny (left) and an hSNP-defined ML phylogeny (right). Phylogenies were generated with IQTREE using automatic model selection based on the Bayesian information criterion (cSNP phylogeny: TVMe+ASC, hSNP phylogeny: K2P, hSNP constrained cSNP phylogeny: TVM+F+ASC+G4). Lineage 4 specimens are outlined in blue. Kendall–Colijn Euclidean distance is indicated at the bottom.

We also observed congruence in the phylogenetic signal present in hSNPs and cSNPs (Fig. 3). When we constrained a cSNP-based phylogeny to the topology of that created with hSNPs, the likelihood was −14 586.23, compared to −12 306.80 for a phylogeny defined only by cSNPs. Constraining the cSNP-based phylogeny to a set of randomly generated tree topologies resulted in much lower likelihood [mean (sd): −25 087.74 (450.46)]. Additionally, we found the cSNP ML tree and the bootstrap replicates were congruent with the hSNP topology based on the AU test (*P*>0.05 for all comparisons). This suggests the phylogenetic signal provided by the hSNPs is much better than expected by chance. However, the KC distance was greater between cSNP and hSNP topologies (109.20) as compared to random [mean (sd): 68.44 (11.96)]. Furthermore, a tanglegram revealed differences between the cSNP and hSNP topologies, indicating that while hSNPs may provide additional information in the case of very closely related infections, they should be used as a complement to cSNP-based analyses and not independently for inferences of transmission.

### Conclusion

We have used WGS data to identify within-host heterogeneity in *M. tuberculosis* amongst patients in British Columbia, Canada. Reliable methods to characterize within-host heterogeneity are needed to incorporate these data into epidemiological investigations. Our data included epidemiologically and/or genetically linked specimens from two *M. tuberculosis* lineages.

We identified sources of bias leading to false positive identification of hSNPs, including excessively clipped reads, read depth, base quality, strand bias, base quality bias, mapping quality bias and read placement bias scores. We also observed concordance between hSNPs, cSNPs and epidemiological data. In one case, a shared variant was identified solely between two epidemiologically linked cases, BC12-Mtb044 and BC13-Mtb142; had hSNPs not been included in the analysis, this would have been missed. This affirms that the inclusion of hSNPs may provide additional resolution to inferences of transmission.

Ultra-deep sequencing may help to identify additional hSNPs and provide further discrimination between transmission events. Furthermore, long read sequencing may be useful in the identification of both cSNPs and hSNPs in repetitive regions of the genome. As suggested by Worby *et al*. [14], we expect hSNPs present in only a small number of very closely genetically related specimens to be the most informative; hSNPs found in a large number of unrelated infections probably reflect systematic analysis errors, or repeated mutation. Ultimately, the potential benefits of this approach will be shown by applying the pipelines we describe here to larger datasets with low strain diversity.

While we have focused here predominantly on biases related to sequencing and bioinformatics approaches, it is important to recognize that all included samples were cultured prior to sequencing. There is currently limited knowledge about the impact of culture on heterogenous alleles. One study from 2017 compared sequencing from sputum with sequencing from MGIT culture using 17 paired samples and found that the median number of hSNPs was the same regardless of sequencing source [40]. However, another study with similar sample size found the opposite, with a significant loss of diversity when sequencing from MGIT culture [41]. Thus, it is possible that sequencing from culture influenced the hSNPs identified. Further investigation is needed on the impact of culture on detection of these heterogeneous alleles.

## Data bibliography

1. DNA sequences have been deposited to NCBI SRA under accession number PRJNA413593 (Table S1). Additional sequences used in this study were also obtained from:
2. . Camus JC, Pryor MJ, Médigue C, Cole ST. Re-annotation of the genome sequence of *Mycobacterium tuberculosis* H37Rv. *Microbiology* [Internet]. 2002;148(1350–0872 LA–eng PT–Journal Article RN–0 (Bacterial Proteins) SB–IM):2967–73. Available from: pm:12368430
3. . Cole ST, Brosch R, Parkhill J, Garnier T, Churcher C *et al*. Deciphering the biology of *Mycobacterium tuberculosis* from the complete genome sequence. *Nature* 1998;393(6685):537–44.
4. . Zhang Y, Chen C, Deng JLH, Pan A, Zhang L et al. Complete genome sequences of *Mycobacterium tuberculosis* strains CCDC5079 and CCDC5080, which belong to the Beijing family. *J Bacteriol* 2011;193(19):5591–2.

## Supplementary Data

Supplementary File 1
