# Supplementary material for: Within-host Mycobacterium tuberculosis diversity and its utility for inferences of transmission
Source: Microb Genom. 2018 Oct 11;4(10):e000217. doi: 10.1099/mgen.0.000217 (PMC6249434; doi:10.1099/mgen.0.000217)
Supplement: Supplementary File 1 [file mgen-4-217-s001.pdf]

# SUPPLEMENTARY METHODS

**Sample collection**

Samples were collected between 2004 and 2014 as part of routine public health investigations in British Columbia, Canada. Samples were chosen from a larger study [1] to represent clusters of similar samples and samples expected to be distantly related. Samples were cultured and genotyped via Mycobacterial Interspersed Repetitive Units-Variable Number of Tandem Repeats (MIRU-VNTR) at the British Columbia Centre for Disease Control (BCCDC) and BC Public Health Laboratory (BCPHL). The BCPHL receives *M. tuberculosis* samples from across British Columbia, and BCCDC maintains the Provincial TB Registry, which includes clinical, demographic, and epidemiological information for each case. Samples were cultured at the BCPHL using the BACTEC MGIT 960 Mycobacterial Detection System (Becton-Dickinson, Franklin Lakes, NJ) on Lowenstein-Jensen (LJ) medium (Bio-Media, Toronto, ON) as described in [1]. In order to capture the full genetic diversity, clean sweeps of LJ slants were taken for sequencing. WGS reads were generated at the BC Genome Sciences Centre using an Illumina HiSeq (Illumina, Inc., San Diego, CA) with 125 bp paired-end reads.

Epidemiological data was collected by local public health units as part of routine investigations and provided by the BCCDC for this work. Data was provided in non-nominal form, consisting of suspected transmission chains and limited drug susceptibility data (i.e., MDR/not-MDR), with no identifying information.

**Genomic Investigation**

FastQ files were trimmed with Trimmomatic v0.36 [2]. Illumina adapters, leading and trailing bases below quality 3, 4-base sliding windows with average quality per base below 15, and any reads less than 36 base pair (bp) were removed. Trimmed FastQ files were assessed for contamination using Kraken v0.10.5-beta with the minikraken_20171019_8GB database [3]. BWA MEM v0.7.15-r1140 [4] was used to align trimmed reads to the H37Rv reference genome [GenBank accession number: NC_000962.3] [5,6] with default settings. Sequence alignment map (SAM) files were sorted and saved as binary alignment map (BAM) files with SAMtools v1.7 using htslib v1.7 [7].

BAM files were visualized using Tablet v1.17.08.17 [8,9] and Interactive Genomics Viewer v2.4.5 [10]. Lineage assignment was done by the visual detection of large sequence polymorphisms (LSPs) [11]. Lineage 4 specimens were characterized by a 7 bp deletion in *pks15/1,* while lineage 2 specimens were characterized by the deletion of RD105 [11]. Trimmed reads from the lineage 2 specimens were additionally aligned to CCDC5079 [GenBank accession number: CP001641] [12]. The same variant calling and filtering pipeline was used for both reference genomes.

Targets for local realignment around small insertions and deletions (indels) were identified with GATK v3.8.0-ge9d806836 [13]. GATK was used for local realignment around indels and read length filtering (minimum read length 100 bases, maximum read length 500 bases, soft clipping at both ends not required). BEDtools v2.26.0 [14] was used to identify regions across the H37Rv genome with no coverage in each BAM file.

SAMtools was used to generate mpileup files from each BAM (maximum depth 1000, minimum mapping quality 30). BCFtools v1.3.1-209-g1618245 using htslib 1.3.2-199-gec1d68e) [7] was used to call variants and generate single specimen variant call format

(VCF) files from mpileups (strand bias (SP) < 60, variant depth on the forward and reverse strands >1, mapping quality (MQ) > 30, base quality (QUAL) > 50, high quality read depth (DP) > 20, base quality recalibration enabled) [15]. For each specimen, regions within 12 base pairs of two high quality SNPs with ≥ 90% variant reads were identified and parsed to BED format using custom Python v2.7 [16] scripts (https://github.com/c2-d2/within-host-diversity). BEDtools was used to filter any variants in these regions on a per-specimen basis. VCF files with variant loci which had failed quality thresholds (SP ≥ 60, variant depth on the forward or reverse strands ≤ 1, mapping quality ≤ 30, base quality ≤ 50, DP ≤ 20) were identified with BCFtools. Regions without coverage in any of the specimens, variant loci failing quality thresholds in any of the specimens, and regions annotated as PE, PPE, or PE_PGRS (PE/PPE) were parsed to BED format using Python scripts and concatenated. BEDtools was used to exclude these regions from the VCF files. SnpEff v4.3t was used to annotate the VCF files using the Mycobacterium_tuberculosis_h37rv and Mycobacterium_tuberculosis_ccdc5079 databases [17].

SNPs were considered consensus SNPs (cSNPs) if at least 90% of the reads supported the variant base and heterogeneous SNPs (hSNPs) if greater than 10% and up to 90% of the reads supported the variant base. SNPs with 10% or less of the reads supporting the variant base were called as reference. Informative SNPs were defined as variants present in at least one, but not all of the 25 specimens.

SNPs associated with drug resistance (Walker et al. 2015 Table S8.1) [18] were used to generate a tab delimited text file. A custom Python script was used to identify the presence of these SNPs in the final VCF files. Both cSNPs and hSNPs were considered for drug resistance.

hSNPs and cSNPs were identified from the final VCF files using Python scripts. Indels were excluded. Figures comparing alignment statistics of SNPs were generated using matplotlib v1.3.1 [19] and seaborn v0.8.1 [20].

As several genomic studies of *M. tuberculosis* have included marking and excluding PCR duplicates in their bioinformatics pipelines [21] we also repeated the above analysis including this step, with the MarkDuplicates command in Picard v2.17.0 [22].

**Phylogenetic analysis**

cSNPs and hSNPs were concatenated into alignments which were used to infer maximum likelihood phylogenies with IQ-TREE v1.6.1 [23] using automatic model selection by Bayesian information criterion (BIC) [24]. Phylogenies were rooted on the reference genome. hSNP alignments were created using IUPAC codes for heterogeneous alleles. Ultrafast bootstrap values with 1000 replicates were calculated to assess branching confidence [25]. Trees were visualized using ggtree v1.10.5 [26] in RStudio v1.1.447 [27]. IQ-TREE was used to generate random phylogenies under a Yule-Harding model with the same tip names as the SNP phylogenies.

Trees were compared using Kendall-Colijn Euclidean distances [28], which were calculated using the treeDist function in the treespace v1.1.2 package with a lambda value of 0 [29]. cSNP and hSNP phylogenies were also compared using the approximately unbiased (AU) test in CONSEL v0.20 [30]. Specifically, we first generated a concatenated tree file containing the cSNP ML tree and corresponding bootstrap replicates. We then used IQ-TREE to estimate the hSNP ML tree and corresponding model parameters, providing it with the cSNP treefile as input (using the -z option). We then calculated the log-likelihoods of these trees using 10,000 RELL replicates [31]. These likelihoods were imported into CONSEL for the

approximately unbiased (AU) test [32]. Tanglegrams used to compare phylogenies were generated using dendextend v1.7.0 [33].

---

# SUPPLEMENTARY RESULTS

**Quality Control**

Samples were collected between 2004 and 2014. More than 98% of reads in each specimen were identified as belonging to *M. tuberculosis* complex (mean [sd]: 99.18 [0.32]) by Kraken v0.10.5-beta with the minikraken_20171019_8GB database [3]. Raw read depths ranged from 112 to 296 (mean [sd]: 181.24 [31.15]). See Tables S1 and S2.

**Marking duplicates**

Amongst all specimens aligned to H37Rv, the mean [SD] % of reads marked as duplicates was 0.71 [0.10]. There was no significant difference in the number of cSNPs (median [IQR]): 1213 [1200-1230] versus 1209 [1196-1226]) or hSNPs (median [IQR]: 15 [14-17] versus 14 [13-16]) comparing analyses without marking duplicates to those with marking duplicates, respectively. As expected, marking duplicates also did not change our epidemiological inferences (Fig. S8). For these reasons, we have not included this step in our primary analysis.

**Alignment induced bias in observed hSNPs**

Both cSNPs and hSNPs were observed more frequently in PE/PPE regions of the genome (Fig. S1). The mean [sd] cSNP frequency was $4.05 \times 10^{-4}$ [0.57] $bp^{-1}$ in PE/PPE

regions and 2.73 × $10^{-4}$ [0.54] bp outside PE/PPE regions. The mean [sd] hSNP frequency was 0.95 × 10-4 [0.11] bp in PE/PPE regions and 0.06 × 10-4 [0.01] bp outside PE/PPE regions. This is indicative of either higher mutation rates [34] or read mapping issues given the repetitive nature of these regions [35,36]. Without long read sequencing data to reliably interrogate these regions [37] and due to a precedent in the field [38], they were removed from further analysis.

**Genomic investigation of serial multi-drug resistant *M. tuberculosis* specimens**

Four specimens in our dataset were collected between 2004 and 2007 from a patient diagnosed with multi-drug resistant (MDR) *M. tuberculosis* (Table S8, Fig. S9). cSNPs associated with resistance to ethambutol (*embB* Met306Val), rifampin (*rpoB* Ser450Leu), and streptomycin (*rpsL* Lys43Arg) were detected in all four specimens. An hSNP associated with ofloxacin and moxifloxacin resistance (*gyrA* Asp94Ala) was observed at 14% supporting variant read frequency but failed our base quality filter (QUAL = 20.29).

An hSNP which was present in the 2004 specimen (*Rv1048c* Arg214His) was not found to be present in later years. Additionally, two novel cSNPs were detected in the 2006 and 2007 specimens. One of these cSNPs was in the *mak* gene which is involved in metabolic pathways, including the trehalose utilization pathway [39,40] and therefore this may represent a compensatory mutation. Both of these cSNPs were unique to these serial specimens.

Furthermore, nine additional novel hSNPs were observed in the 2007 specimen. Of these, all were in annotated regions of the genome. Five of these had functional annotations including hSNPs in a GTP-binding translation elongation factor (*typA*), a cold-shock protein

(*deaD*), a polyprenol monophosphomannose synthase (*ppm1*), a fatty-acid-CoA synthetase (*fad17*), and a chromosome partitioning protein (*parA*).

**Inferences robust to choice of reference genome**

The 20 lineage 2 specimens in our dataset were also aligned to a reference genome from lineage 2. CCDC5079 is from a drug-sensitive Beijing-family isolate sampled from a patient with secondary pulmonary tuberculosis in Fujian Province, China in 2004 [12]. Using this reference resulted in smaller differences in depth of coverage between cSNPs and hSNPs compared to when H37Rv was used as a reference. For CDC5079, the mean [sd] depth of cSNPs and hSNPs were 156.78 [46.68] and 165.42 [69.22], respectively, while for H37Rv, the mean [sd] depth of cSNPs was 169.00 [31.45] and hSNPs was 300.87 [65.01]. Given that 20 out of the 25 specimens were lineage 2, this suggests that differences in depth using the H37Rv reference may be due to structural differences between the lineages leading to alignment error. The CCDC5079 alignment exhibited similar biases to the H37Rv alignment in the other statistics (Fig.  S5, Table S6). More investigation is therefore needed to determine the importance of the choice of reference genome when identifying hSNPs from short-read data.

The median [sd] distance between lineage 2 specimens and CCDC5079 was 205 [55.68]. Compared to this reference, there were a total of 1457 SNP loci (1375 cSNP loci, 87 hSNP loci (48 informative hSNP loci)). Consistent with previous work in the field [21], pairwise cSNP distances between lineage 2 specimens were not significantly affected by the choice of reference; the mean pairwise cSNP distance between lineage 2 specimens was 183.04 (sd [range]: 55.68 [1-261]) when aligned to CCDC5079 vs. 178.45 (sd [range]: 54.20 [0-259]) when aligned to H37Rv.

The maximum likelihood phylogeny of these specimens is shown in Fig. S6. The clustering of epidemiologically linked specimens as well as the variation over time in the serial specimens is consistent with the H37Rv-based analysis. In contrast to the H37Rv alignment, a cSNP which was present in the 2004, 2006, and 2007 serial specimens was called as an hSNP in the 2005 specimen. However, this position was located only 16 bp downstream of a PPE gene and may potentially be a false positive due to alignment issues in PE/PPE regions.

A unique feature of the CCDC5079 alignment is a set of four informative hSNP loci which are SNPs in nearly all of the specimens (Table S7). Four of these positions are located in a hypothetical protein (CDC5079_1896). Further inspection revealed a total of 40 variant positions in a 4579 bp region of this gene. 38 of these loci were SNPs in all 20 lineage 2 specimens. While this gene was not annotated, a Blastp [41,42] search revealed 90% protein identify to polyketide synthase *pks12*. This gene has been shown to harbor significant diversity across strains of *M. tuberculosis* [43] and is involved in the production of phospholipid T cell antigens [44].

# REFERENCES


[1] Guthrie JL, Kong C, Roth D, Jorgensen D, Rodrigues M, Hoang L, et al. Molecular Epidemiology of Tuberculosis in British Columbia, Canada: A 10-Year Retrospective Study. Clin Infect Dis 2018;66:849–56. doi:10.1093/cid/cix906.

[2] Bolger AM, Lohse M, Usadel B. Trimmomatic: A flexible trimmer for Illumina sequence data. Bioinformatics 2014;30:2114–20. doi:10.1093/bioinformatics/btu170.

[3] Wood DE, Salzberg SL. Kraken: Ultrafast metagenomic sequence classification using exact alignments. Genome Biol 2014;15. doi:10.1186/gb-2014-15-3-r46.

[4] Li H, Durbin R. Fast and accurate short read alignment with Burrows-Wheeler transform. Bioinformatics 2009;25:1754–60. doi:10.1093/bioinformatics/btp324.

[5] Camus JC, Pryor MJ, Médigue C, Cole ST. Re-annotation of the genome sequence of Mycobacterium tuberculosis H37Rv. Microbiology 2002;148:2967–73. doi:10.1099/00221287-148-10-2967.

[6] Cole ST, Brosch R, Parkhill J, Garnier T, Churcher C, Harris D, et al. Deciphering the biology of mycobacterium tuberculosis from the complete genome sequence. Nature 1998;393:537–44. doi:10.1038/31159.

[7] Li H, Handsaker B, Wysoker A, Fennell T, Ruan J, Homer N, et al. The Sequence Alignment/Map format and SAMtools. Bioinformatics 2009;25:2078–9. doi:10.1093/bioinformatics/btp352.

[8] Milne I, Bayer M, Cardle L, Shaw P, Stephen G, Wright F, et al. Tablet-next generation sequence assembly visualization. Bioinformatics 2009;26:401–2. doi:10.1093/bioinformatics/btp666.

[9] Milne I, Stephen G, Bayer M, Cock PJA, Pritchard L, Cardle L, et al. Using tablet for visual exploration of second-generation sequencing data. Brief Bioinform 2013;14:193–202. doi:10.1093/bib/bbs012.

[10] Thorvaldsdóttir H, Robinson JT, Mesirov JP. Integrative Genomics Viewer (IGV): High-performance genomics data visualization and exploration. Brief Bioinform 2013;14:178–92. doi:10.1093/bib/bbs017.

[11] Gagneux S, DeRiemer K, Van T, Kato-Maeda M, de Jong BC, Narayanan S, et al. Variable host-pathogen compatibility in Mycobacterium tuberculosis. Proc Natl Acad Sci 2006;103:2869–73. doi:10.1073/pnas.0511240103.

[12] Zhang Y, Chen C, Deng JLH, Pan A, Zhang L, Zhao X, et al. Complete genome sequences of Mycobacterium tuberculosis Strains CCDC5079 and CCDC5080, which belong to the Beijing family. J Bacteriol 2011;193:5591–2. doi:10.1128/JB.05452-11.

[13] Auwera GA Van Der, Carneiro MO, Hartl C, Poplin R, Levy-moonshine A, Jordan T, et al. From FastQ data to high confidence variant calls: the Genome Analysis Toolkit best practices pipeline. vol. 11. 2014. doi:10.1002/0471250953.bi1110s43.From.

[14] Quinlan AR, Hall IM. BEDTools: A flexible suite of utilities for comparing genomic features. Bioinformatics 2010;26:841–2. doi:10.1093/bioinformatics/btq033.

[15] Li H. Improving SNP discovery by base alignment quality. Bioinformatics 2011;27:1157–8. doi:10.1093/bioinformatics/btr076.

[16] Python Software Foundation. Python Language Reference, version 2.7. Python Softw Found 2013:Version 3.03., http://www.python.org. doi:https://www.python.org/.

[17] Cingolani P, Platts A, Wang LL, Coon M, Nguyen T, Wang L, et al. A program for annotating and predicting the effects of single nucleotide polymorphisms, SnpEff. Fly (Austin) 2012;6:80–92. doi:10.4161/fly.19695.

[18] Walker TM, Kohl TA, Omar S V., Hedge J, Del Ojo Elias C, Bradley P, et al. Whole-genome sequencing for prediction of Mycobacterium tuberculosis drug susceptibility and resistance: A retrospective cohort study. Lancet Infect Dis 2015;15:1193–202. doi:10.1016/S1473-3099(15)00062-6.
[19] Hunter JD. Matplotlib: A 2D graphics environment. Comput Sci Eng 2007;9:99–104. doi:10.1109/MCSE.2007.55.
[20] Waskom M, Botvinnik O, O'Kane D, Hobson P, Lukauskas S, Gemperline DC, et al. mwaskom/seaborn: v0.8.1 (September 2017) 2017. doi:10.5281/ZENODO.883859.
[21] Lee RS, Behr MA. Does choice matter? reference-based alignment for molecular epidemiology of tuberculosis. J Clin Microbiol 2016;54:1891–5. doi:10.1128/JCM.00364-16.
[22] Broad Institute. Picard tools. Https://BroadinstituteGithubIo/Picard/ 2016.
[23] Nguyen LT, Schmidt HA, Von Haeseler A, Minh BQ. IQ-TREE: A fast and effective stochastic algorithm for estimating maximum-likelihood phylogenies. Mol Biol Evol 2015;32:268–74. doi:10.1093/molbev/msu300.
[24] Kalyaanamoorthy S, Minh BQ, Wong TKF, Von Haeseler A, Jermiin LS. ModelFinder: Fast model selection for accurate phylogenetic estimates. Nat Methods 2017;14:587–9. doi:10.1038/nmeth.4285.
[25] Hoang DT, Chernomor O, Von Haeseler A, Minh BQ, Vinh LS. UFBoot2: Improving the ultrafast bootstrap approximation. Mol Biol Evol 2018;35:518–22. doi:10.1093/molbev/msx281.
[26] Yu G, Smith DK, Zhu H, Guan Y, Lam TTY. Ggtree: an R Package for Visualization and Annotation of Phylogenetic Trees With Their Covariates and Other Associated Data. Methods Ecol Evol 2017;8:28–36. doi:10.1111/2041-210X.12628.
[27] RStudio Team. RStudio: Integrated Development for R.tle 2015.
[28] Kendall M, Colijn C. Mapping Phylogenetic Trees to Reveal Distinct Patterns of Evolution. Mol Biol Evol 2016;33:2735–43. doi:10.1093/molbev/msw124.
[29] Jombart T, Kendall M, Almagro-Garcia J, Colijn C. treespace: Statistical exploration of landscapes of phylogenetic trees. Mol Ecol Resour 2017;17:1385–92. doi:10.1111/1755-0998.12676.
[30] Shimodaira H, Hasegawa M. CONSEL: for assessing the confidence of phylogenetic tree selection. Bioinformatics 2001;17:1246–7. doi:10.1093/bioinformatics/17.12.1246.
[31] Kishino H, Miyata T, Hasegawa M. Maximum likelihood inference of protein phylogeny and the origin of chloroplasts. J Mol Evol 1990;31:151–60. doi:10.1007/BF02109483.
[32] Shimodaira H. An approximately unbiased test of phylogenetic tree selection. Syst Biol 2002;51:492–508. doi:10.1080/10635150290069913.
[33] Galili T. dendextend: An R package for visualizing, adjusting and comparing trees of hierarchical clustering. Bioinformatics 2015;31:3718–20. doi:10.1093/bioinformatics/btv428.
[34] McEvoy CRE, Cloete R, Müller B, Schürch AC, van Helden PD, Gagneux S, et al. Comparative analysis of mycobacterium tuberculosis pe and ppe genes reveals high sequence variation and an apparent absence of selective constraints. PLoS One 2012;7. doi:10.1371/journal.pone.0030593.
[35] Comas I, Chakravartti J, Small PM, Galagan J, Niemann S, Kremer K, et al. Human T cell epitopes of mycobacterium tubercolsis are evolutionarily hyperconserved. Nat Genet

2010;42:498–503. doi:10.1038/ng.590.Human.

[36] Roetzer A, Diel R, Kohl TA, Rückert C, Nübel U, Blom J, et al. Whole Genome Sequencing versus Traditional Genotyping for Investigation of a Mycobacterium tuberculosis Outbreak: A Longitudinal Molecular Epidemiological Study. PLoS Med 2013;10. doi:10.1371/journal.pmed.1001387.

[37] Bainomugisa A, Duarte T, Lavu E, Pandey S, Coulter C, Marais B, et al. A complete nanonpore-only assembly of an XDR Mycobacterium tuberculosis Beijing lineage strain identifies novel genetic variation in repetitive PE/PPE gene regions. BioRxiv 2018:256719. doi:10.1101/256719.

[38] Comas I, Coscolla M, Luo T, Borrell S, Holt KE, Kato-Maeda M, et al. Out-of-Africa migration and Neolithic coexpansion of Mycobacterium tuberculosis with modern humans. Nat Genet 2013;45:1176–82. doi:10.1038/ng.2744.

[39] Mendes V, Maranha A, Lamosa P, da Costa MS, Empadinhas N. Biochemical characterization of the maltokinase from Mycobacterium bovis BCG. BMC Biochem 2010;11:21. doi:10.1186/1471-2091-11-21.

[40] Fraga J, Maranha A, Mendes V, Pereira PJB, Empadinhas N, Macedo-Ribeiro S. Structure of mycobacterial maltokinase, the missing link in the essential GlgE-pathway. Sci Rep 2015;5:8026. doi:10.1038/srep08026.

[41] Altschul SF, Gish W, Miller W, Myers EW, Lipman DJ. Basic local alignment search tool. J Mol Biol 1990;215:403–10. doi:10.1016/S0022-2836(05)80360-2.

[42] Gish W, States DJ. Identification of protein coding regions by database similarity search. Nat Genet 1993;3:266–72. doi:10.1038/ng0393-266.

[43] Hershberg R, Lipatov M, Small PM, Sheffer H, Niemann S, Homolka S, et al. High functional diversity in Mycobacterium tuberculosis driven by genetic drift and human demography. PLoS Biol 2008;6:2658–71. doi:10.1371/journal.pbio.0060311.

[44] Matsunaga I, Bhatt A, Young DC, Cheng T-Y, Eyles SJ, Besra GS, et al. Mycobacterium tuberculosis pks12 produces a novel polyketide presented by CD1c to T cells. J Exp Med 2004;200:1559–69. doi:10.1084/jem.20041429.

## SUPPLEMENTARY FIGURES AND TABLES

**Fig. S1.** Frequency of consensus SNPs (cSNPs) (left) and heterogeneous SNPs (hSNPs) (right) in non-PE/PPE regions and in PE/PPE regions of the H37RV reference. Both cSNPs and hSNPs are observed more frequently in PE/PPE regions than in the rest of the genome.

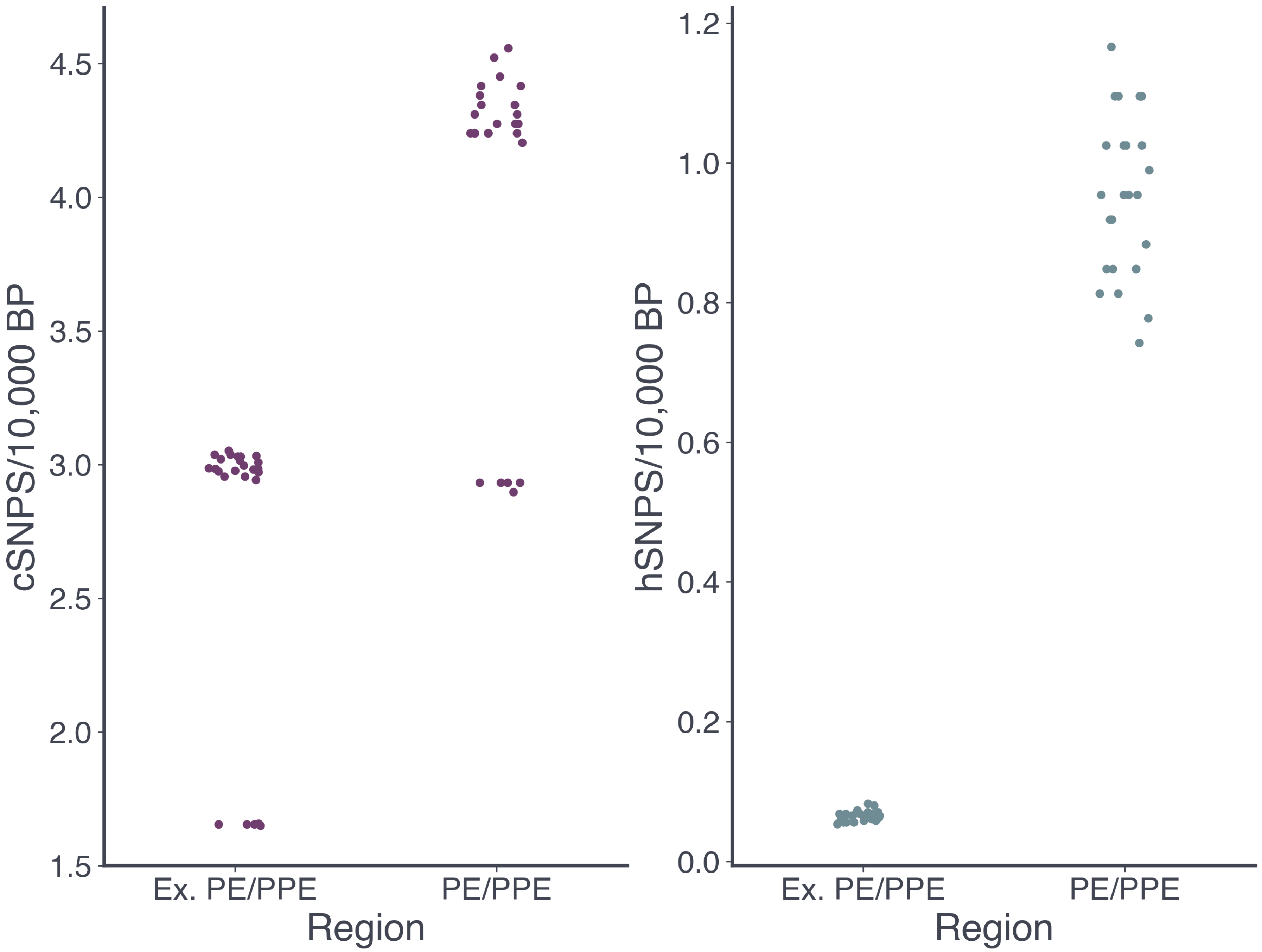

cSNPS/10,000 BP
4.5
4.0
3.5
3.0
2.5
2.0
1.5
Ex. PE/PPE
PE/PPE
Region
hSNPS/10,000 BP
1.2
1.0
0.8
0.6
0.4
0.2
0.0
Ex. PE/PPE
PE/PPE
Region

**Fig. S2.** Distribution of Phred-scaled strand bias p-values comparing consensus SNPs (cSNPs) (purple) to heterogeneous SNPs (hSNPs) (green) following initial filtering protocol (mapping quality > 30, base quality > 50, high quality depth > 20) and exclusion of SNPs in PE/PPE regions. The wider distribution of SP scores amongst hSNPs indicates greater strand bias (variant reads disproportionately supported by either the forward or reverse strands). The maximum observed cSNP SP score was 58.0. Frequency is normalized to 1 to account for different number of observed cSNPs and hSNPs.

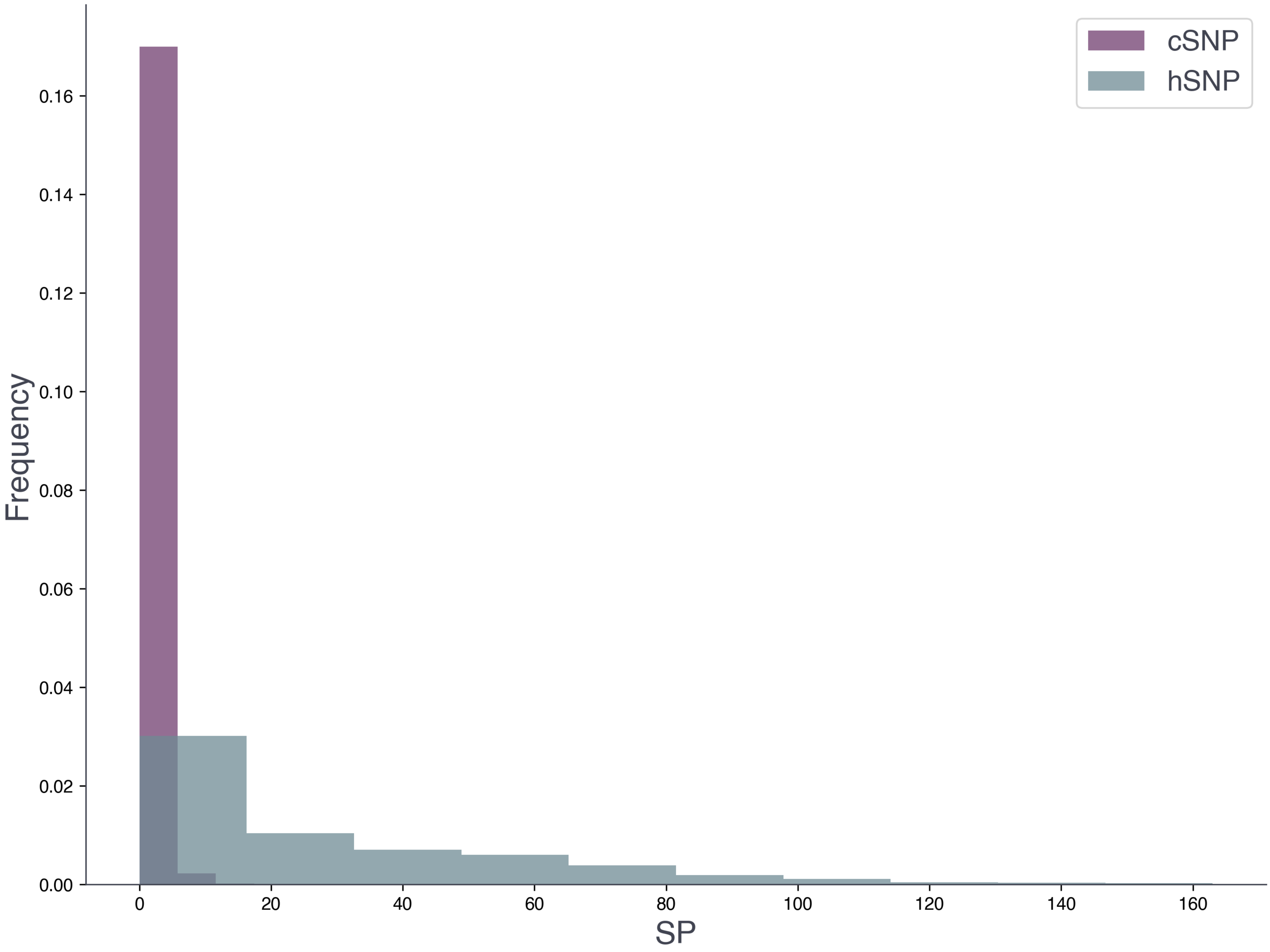

cSNP
hSNP
Frequency
0.00
0.02
0.04
0.06
0.08
0.10
0.12
0.14
0.16
0
20
40
60
80
100
120
140
160
SP

**Fig. S3.** Tablet visualization of read pileups at variant sites (in pink) in which the majority of reads supporting the variant call were clipped (indicated by a red bar) during alignment. Reference reads are indicated in dark gray and the lighter grey represents regions where there are no reads. The blue distribution above the read pileup indicates the read depth. Each visualization is titled with the specimen name and position in the H37Rv alignment.

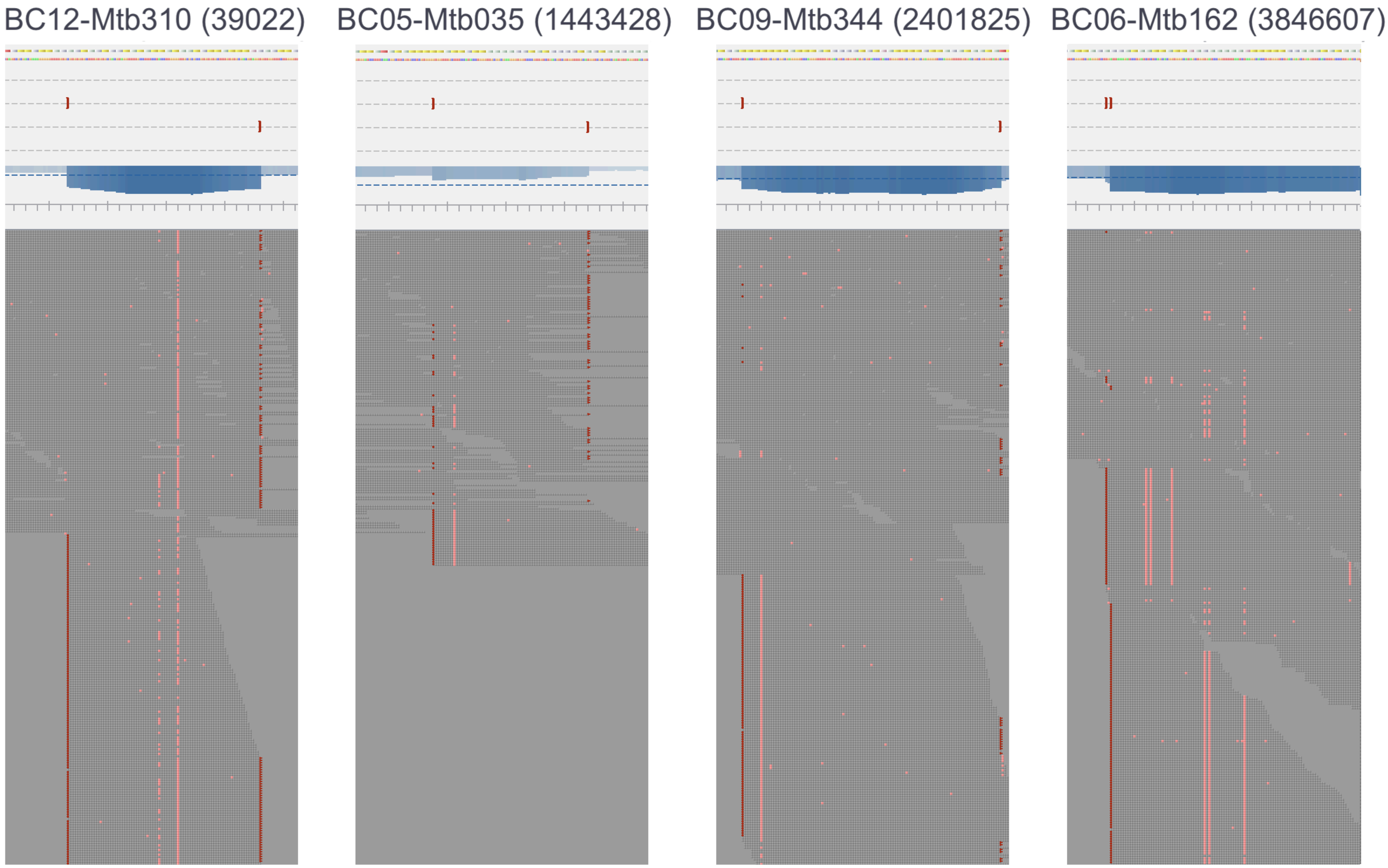
BC12-Mtb310 (39022)
BC05-Mtb035 (1443428)
BC09-Mtb344 (2401825)
BC06-Mtb162 (3846607)

**Fig. S4.** Distribution of heterogeneous SNP (hSNP) presence amongst the 25 specimens included in the study. This considers only variants which are hSNPs in at least one specimen. The presence of a variant as both an hSNP or a consensus SNP (cSNP) is counted as an occurrence. The majority of hSNPs are present only in only one specimen. Peaks are also observed at five specimens, and 20 specimens representing hSNPs shared between all specimens belonging to the same lineage. hSNPs present in all 25 specimens are defined as non-informative.

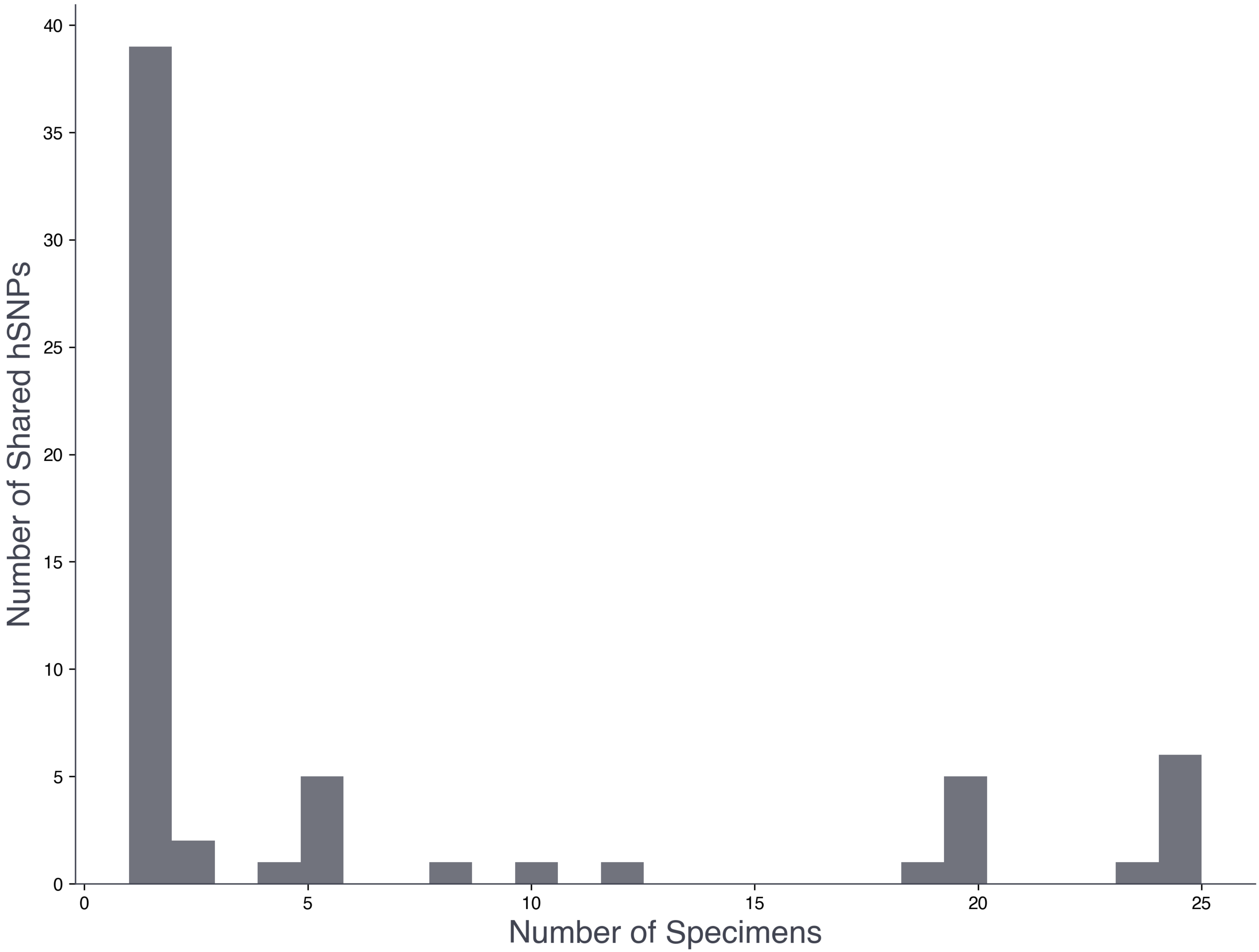

Number of Shared hSNPs
40
35
30
25
20
15
10
5
0
0
5
10
15
20
25
Number of Specimens

**Fig. S5.** (a) Distribution of high quality read depths at consensus SNP (cSNP) (purple) and heterogeneous SNP (hSNP) (green) sites amongst lineage 2 specimens after mapping to the CCDC5079 reference. Read depths are more comparable comparing cSNPs and hSNPs than in the full dataset after mapping to the H37Rv reference (Fig. S4(a)). (b) Recalibrated base quality scores comparing cSNPs (purple) to hSNP (green). hSNPs tend to have lower base quality scores compared to cSNPs. (c) Average mapping quality scores comparing cSNPs (purple) and hSNPs (green). The majority of SNPs have mapping quality scores of 60.

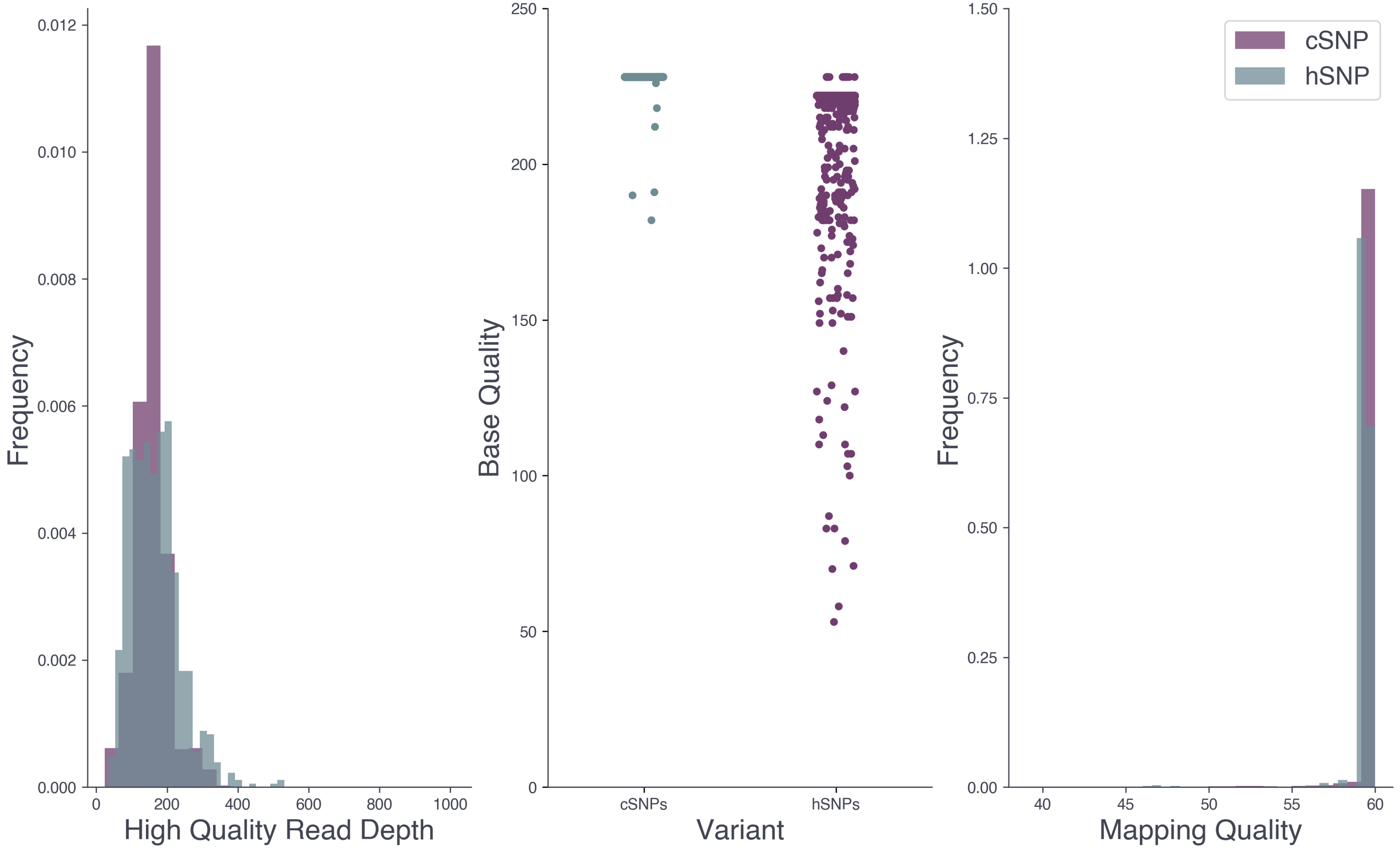
0.012
0.010
0.008
0.006
0.004
0.002
0.000
Frequency
0
200
400
600
800
1000
High Quality Read Depth
250
200
150
100
50
0
Base Quality
cSNPs
hSNPs
Variant
1.50
1.25
1.00
0.75
0.50
0.25
0.00
Frequency
40
45
50
55
60
Mapping Quality
cSNP
hSNP

**Fig. S6.** Maximum likelihood (ML) phylogeny generated using alignment of 1375 consensus SNPs (cSNPs) amongst the 20 lineage 2 specimens, rooted on the CCDC5079 reference (REF). TVMe+ASC was identified as the best-fit model by Bayesian information criterion. Ultrafast bootstrap support values are annotated in blue (support values >95% indicate high confidence) and epidemiological data provided by the BCCDC are labeled in accordance with Fig. 1. Branches without annotation are not epidemiologically linked to other cases in the dataset. Each column at right represents an informative heterogeneous SNP (hSNP) as compared to CCDC5079, ordered by position in the genome and colored by supporting variant read frequency.

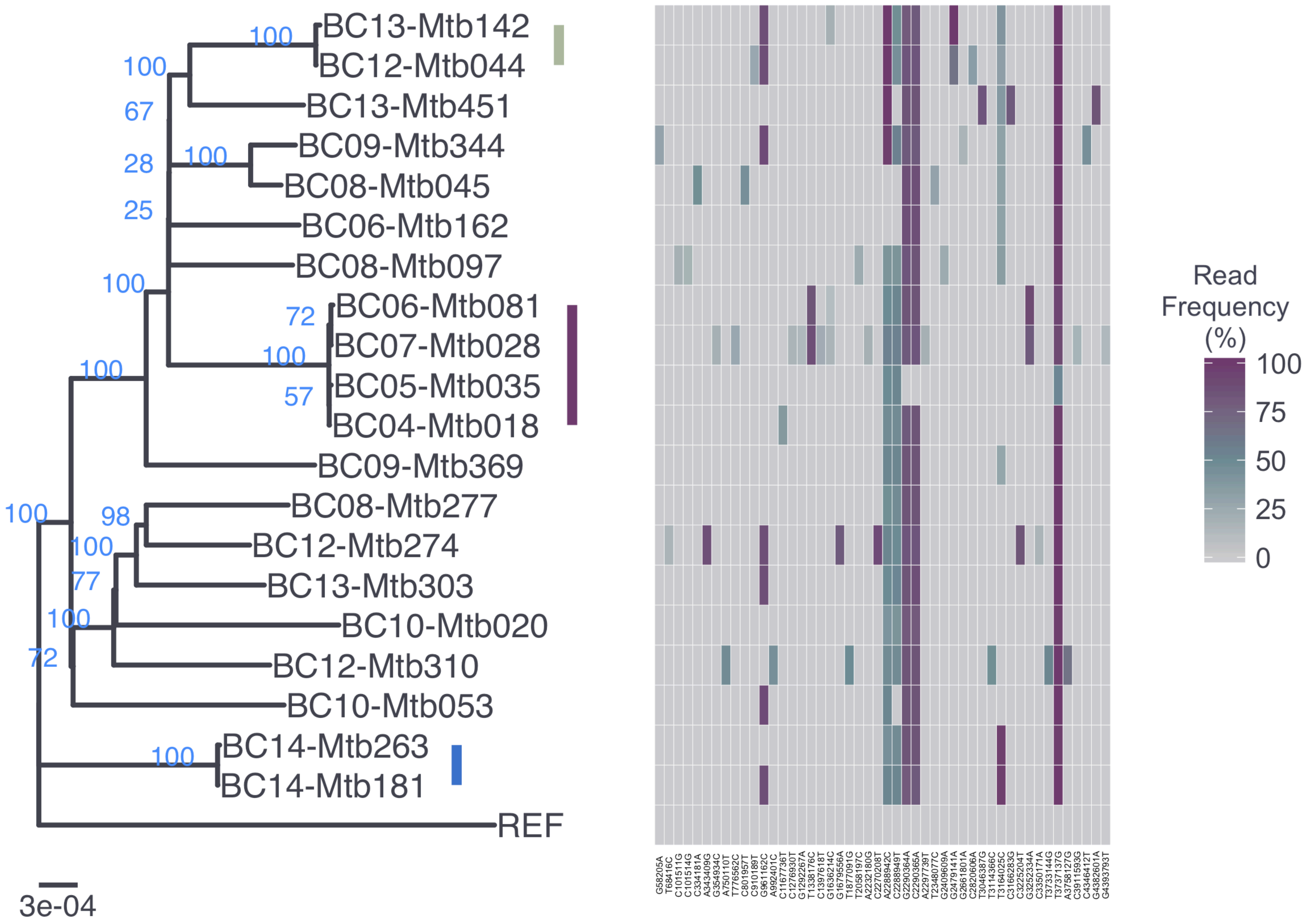
BC13-Mtb142
BC12-Mtb044
BC13-Mtb451
BC09-Mtb344
BC08-Mtb045
BC06-Mtb162
BC08-Mtb097
BC06-Mtb081
BC07-Mtb028
BC05-Mtb035
BC04-Mtb018
BC09-Mtb369
BC08-Mtb277
BC12-Mtb274
BC13-Mtb303
BC10-Mtb020
BC12-Mtb310
BC10-Mtb053
BC14-Mtb263
BC14-Mtb181
REF
100
100
67
28
100
25
100
72
100
100
57
100
98
100
77
100
72
100
3e-04
G58205A
T68416C
C101511G
C101514G
C334181A
A343409G
G354934C
A750110T
T776562C
C801957T
C910189T
G961162C
A992401C
C1167736T
C1276930T
G1292267A
T1338176C
C1397618T
G1636214C
G1679556A
T1877091G
T2058197C
A2232180G
C2270208T
A2288942C
C2288949T
G2290364A
C2290365A
A2297739T
T2348077C
G2409609A
G2479141A
G2661801A
C2820606A
T3046387G
T3114366C
T3164025C
C3166283G
C3225204T
G3252334A
C3350171A
T3733144G
T3737137G
A3758127G
C3911593G
C4346412T
G4382601A
G4393793T
Read Frequency (%)
100
75
50
25
0

**Fig. S7.** (a) Distribution of high quality read depths at consensus SNP (cSNP) (purple) and heterogeneous SNP (hSNP) (green) sites. In general, hSNPs tend to occur at loci with higher read depths (mean [sd]: 232.84 [88.17]) than cSNPs (mean[sd]: 161.84 [41.34])). Frequency is normalized to 1 to account for different number of observed cSNPs and hSNPs. (b) Recalibrated base quality scores comparing cSNPs (purple) and hSNPs (green). Mean base quality scores are comparable (228.00 v. 209.13 respectively), however, there are more lower quality hSNPs (sd: 0.36 v. 34.66, respectively). (c) Average mapping quality scores comparing cSNPs (purple) and hSNPs (green). The distribution summary statistics are similar (mean [sd]: 59.71 [1.95] v. 59.42 [1.57], respectively). Frequency is normalized to 1 to account for different number of observed cSNPs and hSNPs.

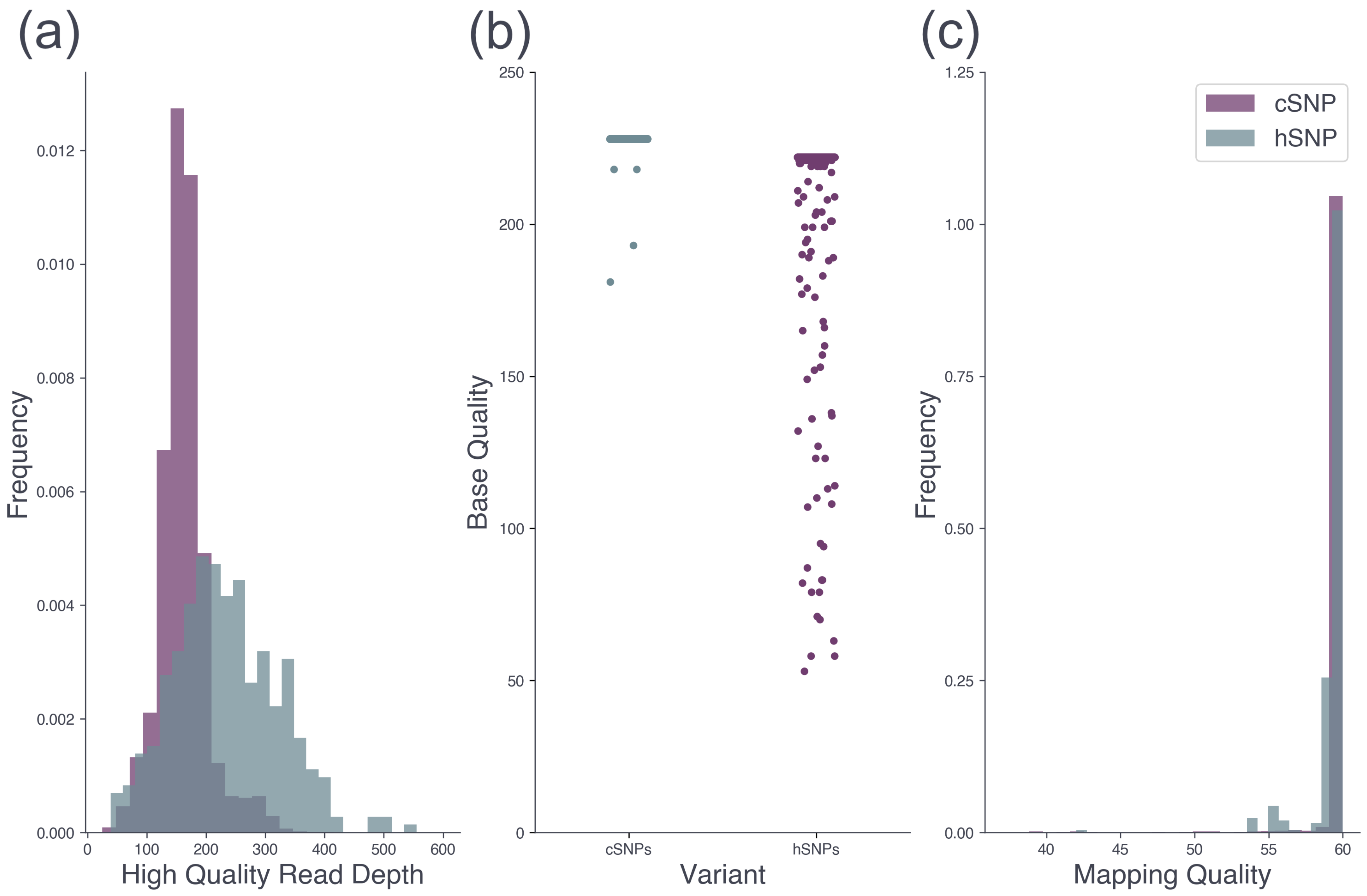
(a)
Frequency
0.000
0.002
0.004
0.006
0.008
0.010
0.012
0
100
200
300
400
500
600
High Quality Read Depth
(b)
Base Quality
0
50
100
150
200
250
cSNPs
hSNPs
Variant
(c)
Frequency
0.00
0.25
0.50
0.75
1.00
1.25
40
45
50
55
60
Mapping Quality
cSNP
hSNP

**Fig. S8.** Maximum likelihood (ML) phylogeny generated using alignment of 2539 consensus SNPs (cSNPs) rooted on the H37RV reference, following the removal of reads marked as PCR duplicates by Picard v2.17.0 [22]. TVMe+ASC was identified as the best-fit model by Bayesian information criterion. Ultrafast bootstrap support values are annotated in blue (support values >95% indicate high confidence) and epidemiological data provided by the BCCDC are labeled. Branches without annotation are not epidemiologically linked to other cases in the dataset. Each column at right represents an informative heterogeneous SNP (hSNP) as compared to H37Rv, ordered by position in the genome and colored by supporting variant read frequency.

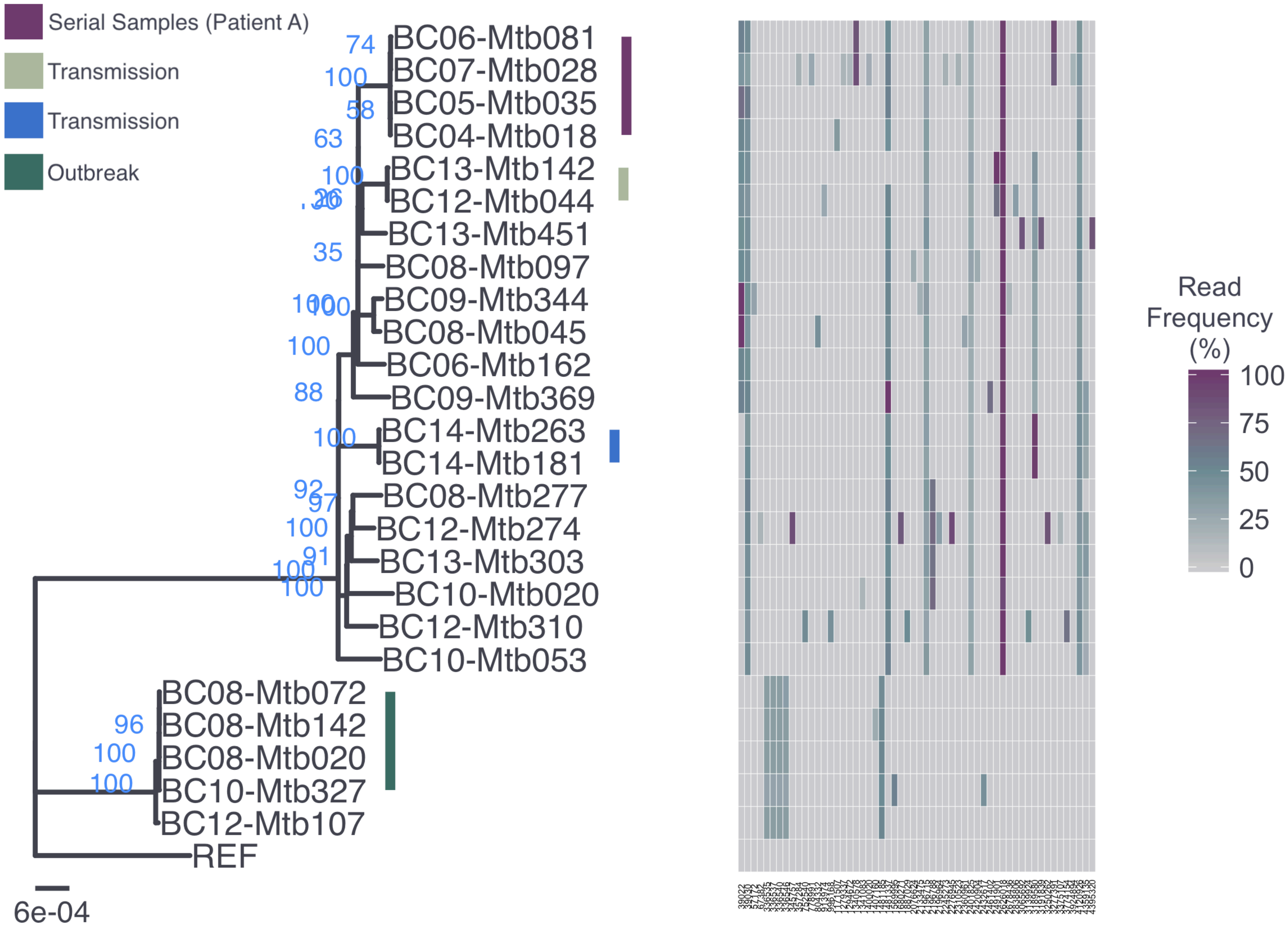

Serial Samples (Patient A)
Transmission
Transmission
Outbreak
BC06-Mtb081
BC07-Mtb028
BC05-Mtb035
BC04-Mtb018
BC13-Mtb142
BC12-Mtb044
BC13-Mtb451
BC08-Mtb097
BC09-Mtb344
BC08-Mtb045
BC06-Mtb162
BC09-Mtb369
BC14-Mtb263
BC14-Mtb181
BC08-Mtb277
BC12-Mtb274
BC13-Mtb303
BC10-Mtb020
BC12-Mtb310
BC10-Mtb053
BC08-Mtb072
BC08-Mtb142
BC08-Mtb020
BC10-Mtb327
BC12-Mtb107
REF
6e-04
Read Frequency (%)
100
75
50
25
0

**Fig. S9.** Informative SNPs in four annual serial specimens from a patient diagnosed with multi-drug resistant (MDR) *M. tuberculosis.* Variants are shown at the X-axis labeled by reference base, position in the H37Rv reference, and the variant base. Sampling year is shown on the Y-axis. Variant read frequencies are represented by shading with green representing 50% variant and 50% reference reads at that position and purple representing 100% variant reads.

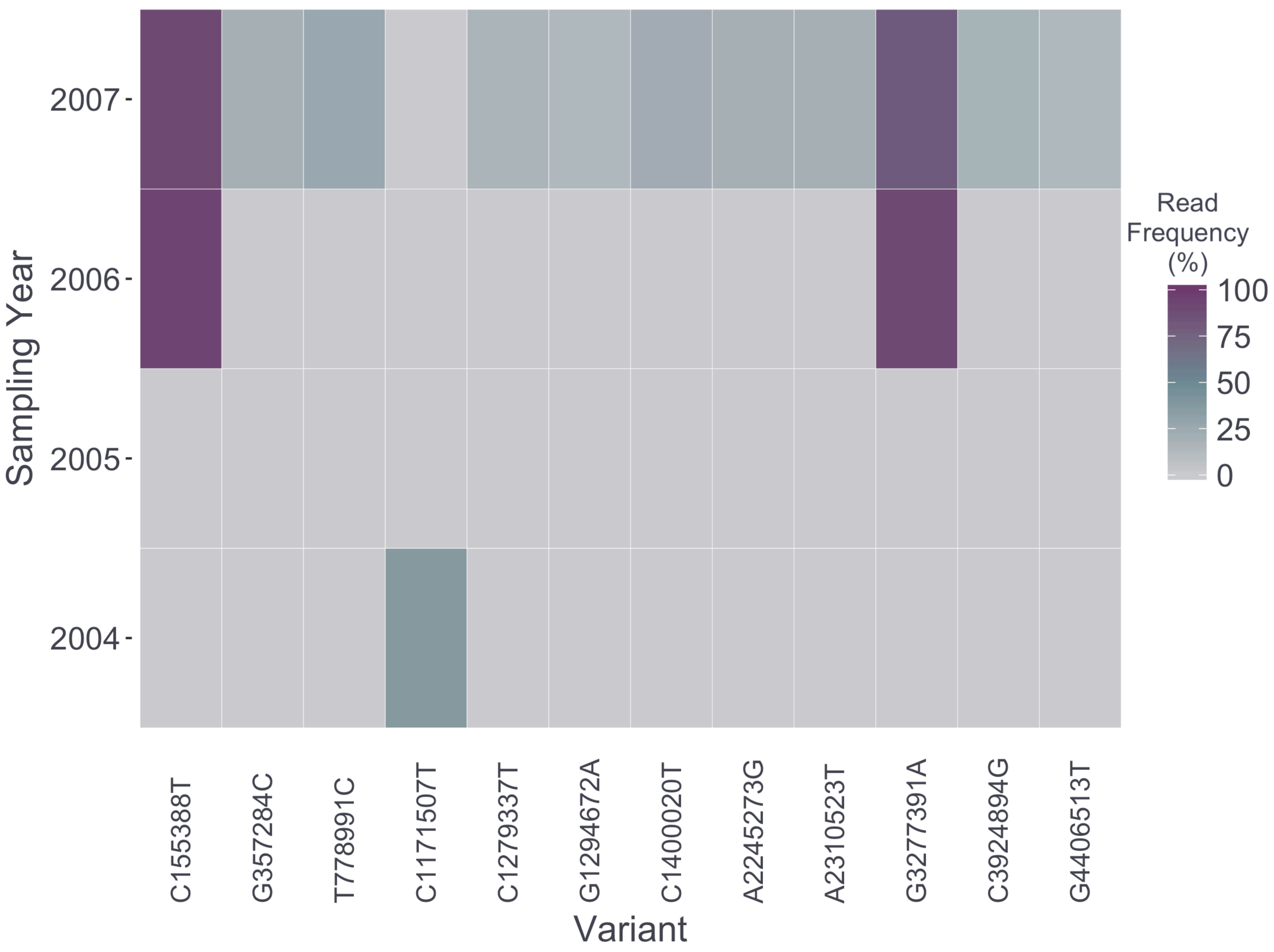

Sampling Year
2007
2006
2005
2004
Variant
C155388T
G357284C
T778991C
C1171507T
C1279337T
G1294672A
C1400020T
A2245273G
A2310523T
G3277391A
C3924894G
G4406513T
Read Frequency (%)
100
75
50
25
0

**Table S1.** Sample characteristics for *M. tuberculosis* specimens

| Sample | Epidemiological Data | MIRU-VNTR Cluster | *In silico M. tuberculosis* lineage* | WGS Drug Resistance Prediction† | Accession Number |
|---|---|---|---|---|---|
| BC04-Mtb018 | 2004 sample (Patient A) | MClust-187 | 2 | EMB, RIF, SM | SRX3490950 |
| BC05-Mtb035 | 2005 sample (Patient A) | MClust-187 | 2 | EMB, RIF, SM | SRX3490874 |
| BC06-Mtb081 | 2006 sample (Patient A) | MClust-187 | 2 | EMB, RIF, SM | SRX3490928 |
| BC06-Mtb162 | No known contact | MClust-187 | 2 | N/A | SRX3265155 |
| BC07-Mtb028 | 2007 sample (Patient A) | MClust-187 | 2 | EMB, RIF, SM | SRX3490687 |
| BC08-Mtb020 | Suspected transmission to BC08-Mtb072, BC08-Mtb142, BC10-Mtb327 | MClust-010 | 4 | N/A | SRX3490842 |
| BC08-Mtb045 | No known contact | MClust-187 | 2 | N/A | SRX3264846 |
| BC08-Mtb072 | Likely infected by BC08-Mtb020 | MClust-010 | 4 | N/A | SRX3490966 |
| BC08-Mtb097 | No known contact | MClust-187 | 2 | N/A | SRX3265123 |
| BC08-Mtb142 | Likely infected by BC08-Mtb020 | MClust-010 | 4 | N/A | SRX3490578 |
| BC08-Mtb277 | No known contact | MClust-187 | 2 | SM | SRX3265071 |
| BC09-Mtb344 | No known contact | MClust-187 | 2 | N/A | SRX3264947 |
| BC09-Mtb369 | No known contact | MClust-187 | 2 | N/A | SRX3264949 |
| BC10-Mtb020 | No known contact | MClust-187 | 2 | N/A | SRX3265087 |
| BC10-Mtb053 | No known contact | MClust-187 | 2 | N/A | SRX3265261 |
| BC10-Mtb327 | Likely infected by BC08-Mtb020 | MClust-010 | 4 | N/A | SRX3490816 |
| BC12-Mtb044 | Suspected transmission to BC13-Mtb142 | MClust-187 | 2 | SM | SRX3491247 |
| BC12-Mtb107 | No known contact | MClust-010 | 4 | N/A | SRX3491200 |
| BC12-Mtb274 | No known contact | MClust-187 | 2 | N/A | SRX3264975 |
| BC12-Mtb310 | No known contact | MClust-187 | 2 | N/A | SRX3265115 |
| BC13-Mtb142 | Likely infected by BC12-Mtb044 | MClust-187 | 2 | SM | SRX3491094 |
| BC13-Mtb303 | No known contact | MClust-187 | 2 | N/A | SRX3264630 |
| BC13-Mtb451 | No known contact | MClust-187 | 2 | N/A | SRX3264658 |
| BC14-Mtb181 | Suspected transmission to BC14-Mtb263 | MClust-187 | 2 | INH, RIF | SRX3491197 |
| BC14-Mtb263 | Likely infected by BC14-Mtb181 | MClust-187 | 2 | INH, RIF | SRX3491162 |

* Lineage 4 identified by a 7 bp deletion in the pks1/15 gene50 and lineage 2 identified by the deletion of RD105 in WGS alignment.51.

† Based on drug resistance mutations from Table S8.1 of Walker et al., 2015 [18].
EMB = ethambutol, MOX = moxifloxacin, OFX = ofloxacin, RIF = rifampin, SM = streptomycin.

**Table S2.** *M. tuberculosis* specimen quality control

| Sample | Collection Date | Average Read Depth* | % Reads MTB Complex (MTB Complex / Total)† |
|---|---|---|---|
| BC04-Mtb018 | 2004 | 183 | 99.49 (3279351/3296172) |
| BC05-Mtb035 | 2005 | 175 | 99.34 (3158805/3179653) |
| BC06-Mtb081 | 2006 | 203 | 99.3 (3673335/3699282) |
| BC06-Mtb162 | 2006 | 173 | 99.29 (3131499/3153921) |
| BC07-Mtb028 | 2007 | 112 | 99.02 (2037398/2057592) |
| BC08-Mtb020 | 2008 | 198 | 99.02 (3596355/3631797) |
| BC08-Mtb045 | 2008 | 188 | 99.1 (3407553/3438558) |
| BC08-Mtb072 | 2008 | 187 | 99.26 (3373798/3399089) |
| BC08-Mtb097 | 2008 | 208 | 99.29 (3774338/3801514) |
| BC08-Mtb142 | 2008 | 185 | 99.36 (3347496/3369180) |
| BC08-Mtb277 | 2008 | 184 | 99.44 (3332624/3351287) |
| BC09-Mtb344 | 2009 | 191 | 98.67 (3459595/3506288) |
| BC09-Mtb369 | 2009 | 165 | 99.46 (2991387/3007699) |
| BC10-Mtb020 | 2010 | 174 | 98.18 (3161524/3220257) |
| BC10-Mtb053 | 2010 | 171 | 99.42 (3101748/3119886) |
| BC10-Mtb327 | 2010 | 203 | 99.48 (3141890/3158271) |
| BC12-Mtb044 | 2012 | 174 | 98.92 (3162958/3197495) |
| BC12-Mtb107 | 2012 | 179 | 98.54 (3242443/3290358) |
| BC12-Mtb274 | 2012 | 153 | 99.10 (2781493/2806713) |
| BC12-Mtb310 | 2012 | 157 | 99.19 (2837279/2860363) |
| BC13-Mtb142 | 2013 | 170 | 99.46 (3061367/3077939) |
| BC13-Mtb303 | 2013 | 296 | 99.14 (5373351/5419856) |
| BC13-Mtb451 | 2013 | 178 | 99.24 (3243153/3268038) |
| BC14-Mtb181 | 2014 | 175 | 99.45 (3181228/3198876) |
| BC14-Mtb263 | 2014 | 149 | 99.43 (2720022/2735569) |

*WGS read depth was assessed based on unfiltered BAM files generated using trimmed reads using SAMtools v1.3.1.

†M. tuberculosis complex content was assessed using Kraken v0.10.5-beta with the minikraken_20171019_8GB database on trimmed reads.

**Table S3.** hSNP call changes following removal of reads with less than 100 bp aligned to H37Rv

| Position | Variant | Sample | DP incl. CR | Variant RF % incl. CR | Variant Call incl. CR | DP excl. CR | Variant RF% excl. CR | Variant Call excl. CR |
|---|---|---|---|---|---|---|---|---|
| 39022 | A39022G | BC04-Mtb018 | 226 | 72% | N/A | 145 | 52% | hSNP |
| | | BC05-Mtb035 | 58 | 83% | N/A | 41 | 68% | hSNP |
| | | BC06-Mtb081 | 228 | 66% | N/A | 138 | 55% | hSNP |
| | | BC06-Mtb162 | 292 | 52% | N/A | 175 | 53% | hSNP |
| | | BC07-Mtb028 | 184 | 70% | N/A | 98 | 41% | hSNP |
| | | BC08-Mtb045 | 160 | 81% | N/A | 90 | 100% | cSNP |
| | | BC08-Mtb097 | 290 | 52% | N/A | 180 | 51% | hSNP |
| | | BC08-Mtb277 | 278 | 19% | N/A | 150 | 0% | REF |
| | | BC09-Mtb344 | 237 | 85% | N/A | 135 | 100% | cSNP |
| | | BC09-Mtb369 | 243 | 55% | N/A | 135 | 54% | hSNP |
| | | BC10-Mtb020 | 293 | 37% | N/A | 167 | 0% | REF |
| | | BC10-Mtb053 | 154 | 16% | N/A | 96 | 0% | REF |
| | | BC12-Mtb044 | 296 | 66% | N/A | 162 | 45% | hSNP |
| | | BC13-Mtb142 | 100 | 76% | N/A | 61 | 49% | hSNP |
| | | BC13-Mtb303 | 319 | 37% | N/A | 170 | 0% | REF |
| | | BC13-Mtb451 | 321 | 69% | N/A | 192 | 47% | hSNP |
| | | BC14-Mtb181 | 253 | 19% | N/A | 137 | 0% | REF |
| 39030 | | BC04-Mtb018 | 228 | 69% | N/A | 144 | 46% | hSNP |
| | | BC05-Mtb035 | 58 | 78% | N/A | 39 | 59% | hSNP |
| | | BC06-Mtb081 | 227 | 64% | N/A | 141 | 48% | hSNP |
| | | BC06-Mtb162 | 294 | 71% | N/A | 176 | 47% | hSNP |
| | | BC07-Mtb028 | 186 | 67% | N/A | 101 | 36% | hSNP |
| | | BC08-Mtb020 | 163 | 100% | N/A | 160 | 0% | REF |
| | | BC08-Mtb045 | 160 | 68% | N/A | 92 | 41% | hSNP |
| | | BC08-Mtb072 | 188 | 100% | N/A | 185 | 0% | REF |
| | | BC08-Mtb097 | 292 | 66% | N/A | 174 | 44% | hSNP |
| | | BC08-Mtb142 | 81 | 100% | N/A | 80 | 0% | REF |
| | | BC08-Mtb277 | 274 | 73% | N/A | 145 | 54% | hSNP |
| | | BC09-Mtb344 | 239 | 69% | N/A | 140 | 46% | hSNP |
| | | BC09-Mtb369 | 243 | 71% | N/A | 134 | 51% | hSNP |
| | | BC10-Mtb020 | 301 | 67% | N/A | 172 | 44% | hSNP |
| | | BC10-Mtb053 | 150 | 71% | N/A | 91 | 47% | hSNP |
| | | BC12-Mtb044 | 295 | 64% | N/A | 162 | 40% | hSNP |
| | | BC12-Mtb274 | 74 | 69% | N/A | 51 | 51% | hSNP |
| | | BC12-Mtb310 | 237 | 64% | N/A | 138 | 40% | hSNP |
| | | BC13-Mtb142 | 101 | 70% | N/A | 63 | 44% | hSNP |
| | | BC13-Mtb303 | 321 | 69% | N/A | 176 | 44% | hSNP |
| | | BC13-Mtb451 | 325 | 69% | N/A | 197 | 44% | hSNP |
| | | BC14-Mtb181 | 254 | 71% | N/A | 138 | 49% | hSNP |
| | | BC14-Mtb263 | 186 | 65% | N/A | 114 | 42% | hSNP |
| 101202 | | BC12-Mtb274 | 150 | 13% | N/A | 147 | 13% | hSNP |
| 336504 | | BC04-Mtb018 | 184 | 99% | cSNP | 71 | 99% | cSNP |
| | | BC05-Mtb035 | 81 | 99% | cSNP | 34 | 97% | cSNP |
| | | BC06-Mtb081 | 80 | 99% | cSNP | 31 | 97% | cSNP |
| | | BC06-Mtb162 | 133 | 100% | cSNP | 54 | 100% | cSNP |
| | | BC07-Mtb028 | 63 | 100% | cSNP | 26 | 100% | cSNP |
| | | BC08-Mtb020 | 422 | 45% | hSNP | 315 | 28% | hSNP |
| | | BC08-Mtb045 | 101 | 100% | cSNP | 46 | 100% | cSNP |
| | | BC08-Mtb072 | 380 | 45% | hSNP | 282 | 26% | hSNP |
| | | BC08-Mtb097 | 161 | 100% | cSNP | 64 | 100% | cSNP |
| | | BC08-Mtb142 | 408 | 45% | hSNP | 294 | 24% | hSNP |
| | | BC08-Mtb277 | 149 | 100% | cSNP | 48 | 100% | cSNP |
| | | BC09-Mtb344 | 155 | 99% | cSNP | 51 | 98% | cSNP |
| | | BC09-Mtb369 | 124 | 98% | cSNP | 56 | 100% | cSNP |
| | | BC10-Mtb020 | 171 | 97% | cSNP | 68 | 93% | cSNP |
| | | BC10-Mtb053 | 157 | 100% | cSNP | 52 | 100% | cSNP |
| | | BC10-Mtb327 | 458 | 40% | hSNP | 273 | 0% | REF |
| | | BC12-Mtb044 | 151 | 98% | cSNP | 57 | 95% | cSNP |
| | | BC12-Mtb107 | 536 | 42% | hSNP | 392 | 22% | hSNP |
| | | BC12-Mtb274 | 139 | 99% | cSNP | 49 | 100% | cSNP |
| | | BC12-Mtb310 | 107 | 99% | cSNP | 53 | 98% | cSNP |
| | | BC13-Mtb142 | 100 | 100% | cSNP | 53 | 100% | cSNP |
| | | BC13-Mtb303 | 201 | 100% | cSNP | 72 | 100% | cSNP |
| | | BC13-Mtb451 | 125 | 99% | cSNP | 41 | 100% | cSNP |
| | | BC14-Mtb181 | 142 | 99% | cSNP | 57 | 98% | cSNP |
| | | BC14-Mtb263 | 135 | 99% | cSNP | 43 | 95% | cSNP |
| 1443428 | | BC04-Mtb018 | 190 | 21% | hSNP | 139 | 19% | N/A |
| | | BC05-Mtb035 | 91 | 19% | hSNP | 59 | 0% | N/A |
| | | BC06-Mtb081 | 188 | 32% | hSNP | 137 | 27% | N/A |
| | | BC06-Mtb162 | 194 | 31% | hSNP | 139 | 26% | N/A |
| | | BC07-Mtb028 | 128 | 34% | hSNP | 93 | 28% | N/A |
| | | BC08-Mtb020 | 172 | 0% | REF | 169 | 0% | N/A |
| | | BC08-Mtb045 | 182 | 30% | hSNP | 131 | 21% | N/A |
| | | BC08-Mtb072 | 160 | 0% | REF | 159 | 0% | N/A |
| | | BC08-Mtb097 | 256 | 26% | hSNP | 195 | 26% | N/A |
| | | BC08-Mtb142 | 133 | 0% | REF | 128 | 0% | N/A |
| | | BC08-Mtb277 | 231 | 29% | hSNP | 164 | 23% | N/A |
| | | BC09-Mtb344 | 235 | 26% | hSNP | 165 | 20% | N/A |
| | | BC09-Mtb369 | 203 | 35% | hSNP | 136 | 27% | N/A |
| | | BC10-Mtb020 | 243 | 31% | hSNP | 161 | 26% | N/A |

| | | | | | | | |
|---|---|---|---|---|---|---|---|
| | BC10-Mtb053 | 173 | 28% | hSNP | 116 | 24% | N/A |
| | BC10-Mtb327 | 184 | 0% | REF | 180 | 0% | N/A |
| | BC12-Mtb044 | 236 | 36% | hSNP | 154 | 24% | N/A |
| | BC12-Mtb107 | 161 | 0% | REF | 160 | 0% | N/A |
| | BC12-Mtb274 | 120 | 33% | hSNP | 88 | 18% | N/A |
| | BC12-Mtb310 | 240 | 29% | hSNP | 166 | 25% | N/A |
| | BC13-Mtb142 | 120 | 27% | hSNP | 93 | 22% | N/A |
| | BC13-Mtb303 | 304 | 29% | hSNP | 213 | 25% | N/A |
| | BC13-Mtb451 | 228 | 24% | hSNP | 151 | 20% | N/A |
| | BC14-Mtb181 | 231 | 35% | hSNP | 157 | 26% | N/A |
| | BC14-Mtb263 | 171 | 22% | hSNP | 118 | 15% | N/A |
| 1472767 | BC09-Mtb344 | 54 | 13% | hSNP | 47 | 0% | REF |
| 1472781 | BC09-Mtb344 | 53 | 13% | hSNP | 46 | 0% | REF |
| 1472793 | BC09-Mtb344 | 56 | 14% | hSNP | 48 | 0% | REF |
| 1472803 | BC09-Mtb344 | 56 | 13% | hSNP | 48 | 0% | REF |
| 1480972 | BC04-Mtb018 | 185 | 58% | N/A | 165 | 65% | hSNP |
| | BC05-Mtb035 | 253 | 60% | N/A | 212 | 70% | hSNP |
| | BC06-Mtb081 | 261 | 61% | N/A | 227 | 69% | hSNP |
| | BC06-Mtb162 | 184 | 60% | N/A | 148 | 72% | hSNP |
| | BC07-Mtb028 | 161 | 61% | N/A | 130 | 72% | hSNP |
| | BC08-Mtb020 | 262 | 63% | N/A | 210 | 77% | hSNP |
| | BC08-Mtb045 | 252 | 60% | N/A | 202 | 74% | hSNP |
| | BC08-Mtb072 | 256 | 59% | N/A | 210 | 69% | hSNP |
| | BC08-Mtb097 | 258 | 52% | N/A | 224 | 60% | hSNP |
| | BC08-Mtb142 | 198 | 58% | N/A | 166 | 68% | hSNP |
| | BC08-Mtb277 | 218 | 51% | N/A | 170 | 66% | hSNP |
| | BC09-Mtb344 | 176 | 54% | N/A | 143 | 66% | hSNP |
| | BC09-Mtb369 | 179 | 59% | N/A | 144 | 74% | hSNP |
| | BC10-Mtb020 | 233 | 64% | N/A | 202 | 73% | hSNP |
| | BC10-Mtb053 | 156 | 56% | N/A | 121 | 71% | hSNP |
| | BC10-Mtb327 | 319 | 60% | N/A | 267 | 71% | hSNP |
| | BC12-Mtb044 | 224 | 63% | N/A | 186 | 75% | hSNP |
| | BC12-Mtb107 | 188 | 56% | N/A | 150 | 68% | hSNP |
| | BC12-Mtb274 | 237 | 59% | N/A | 192 | 70% | hSNP |
| | BC12-Mtb310 | 219 | 64% | N/A | 189 | 74% | hSNP |
| | BC13-Mtb142 | 255 | 62% | N/A | 219 | 72% | hSNP |
| | BC13-Mtb303 | 427 | 62% | N/A | 356 | 73% | hSNP |
| | BC13-Mtb451 | 265 | 60% | N/A | 219 | 72% | hSNP |
| | BC14-Mtb181 | 156 | 63% | N/A | 125 | 77% | hSNP |
| | BC14-Mtb263 | 205 | 58% | N/A | 158 | 74% | hSNP |
| 2133475 | BC09-Mtb344 | 74 | 100% | INDEL | 151 | 21% | hSNP |
| 2266517 | BC04-Mtb018 | 271 | 37% | N/A | 262 | 36% | hSNP |
| | BC05-Mtb035 | 322 | 38% | N/A | 304 | 36% | hSNP |
| | BC06-Mtb081 | 343 | 44% | N/A | 326 | 42% | hSNP |
| | BC06-Mtb162 | 256 | 41% | N/A | 243 | 39% | hSNP |
| | BC07-Mtb028 | 224 | 45% | N/A | 210 | 42% | hSNP |
| | BC08-Mtb020 | 345 | 39% | N/A | 322 | 38% | hSNP |
| | BC08-Mtb045 | 282 | 37% | N/A | 265 | 33% | hSNP |
| | BC08-Mtb072 | 329 | 38% | N/A | 317 | 37% | hSNP |
| | BC08-Mtb097 | 331 | 44% | N/A | 314 | 41% | hSNP |
| | BC08-Mtb142 | 248 | 40% | N/A | 231 | 37% | hSNP |
| | BC08-Mtb277 | 217 | 26% | N/A | 206 | 23% | hSNP |
| | BC09-Mtb344 | 280 | 36% | N/A | 267 | 35% | hSNP |
| | BC09-Mtb369 | 254 | 30% | N/A | 240 | 26% | hSNP |
| | BC10-Mtb020 | 252 | 45% | N/A | 231 | 43% | hSNP |
| | BC10-Mtb053 | 221 | 29% | N/A | 209 | 26% | hSNP |
| | BC10-Mtb327 | 267 | 33% | N/A | 249 | 28% | hSNP |
| | BC12-Mtb044 | 235 | 34% | N/A | 218 | 32% | hSNP |
| | BC12-Mtb107 | 274 | 31% | N/A | 260 | 29% | hSNP |
| | BC12-Mtb274 | 224 | 30% | N/A | 214 | 27% | hSNP |
| | BC12-Mtb310 | 219 | 34% | N/A | 204 | 31% | hSNP |
| | BC13-Mtb142 | 277 | 38% | N/A | 255 | 33% | hSNP |
| | BC13-Mtb303 | 456 | 31% | N/A | 424 | 28% | hSNP |
| | BC13-Mtb451 | 313 | 40% | N/A | 289 | 36% | hSNP |
| | BC14-Mtb181 | 187 | 32% | N/A | 168 | 25% | hSNP |
| | BC14-Mtb263 | 183 | 32% | N/A | 171 | 29% | hSNP |
| 2266598 | BC10-Mtb020 | 296 | 53% | N/A | 255 | 38% | hSNP |
| | BC10-Mtb053 | 230 | 32% | N/A | 295 | 42% | hSNP |
| | BC10-Mtb327 | 276 | 37% | N/A | 317 | 43% | hSNP |
| | BC12-Mtb044 | 252 | 35% | N/A | 247 | 39% | hSNP |
| | BC12-Mtb107 | 274 | 36% | N/A | 199 | 39% | hSNP |
| | BC12-Mtb274 | 237 | 33% | N/A | 311 | 35% | hSNP |
| | BC12-Mtb310 | 200 | 39% | N/A | 287 | 32% | hSNP |
| | BC13-Mtb142 | 292 | 40% | N/A | 354 | 40% | hSNP |
| | BC13-Mtb303 | 539 | 33% | N/A | 297 | 43% | hSNP |
| | BC13-Mtb451 | 330 | 40% | N/A | 236 | 31% | hSNP |
| | BC14-Mtb181 | 202 | 30% | N/A | 208 | 20% | hSNP |
| | BC14-Mtb263 | 180 | 36% | N/A | 229 | 33% | hSNP |
| | BC04-Mtb018 | 278 | 42% | N/A | 214 | 21% | hSNP |
| | BC05-Mtb035 | 321 | 46% | N/A | 251 | 46% | hSNP |
| | BC06-Mtb081 | 342 | 46% | N/A | 205 | 27% | hSNP |
| | BC06-Mtb162 | 275 | 44% | N/A | 244 | 30% | hSNP |

| | | | | | | | |
|---|---|---|---|---|---|---|---|
| | BC07-Mtb028 | 226 | 46% | N/A | 228 | 29% | hSNP |
| | BC08-Mtb020 | 345 | 41% | N/A | 251 | 31% | hSNP |
| | BC08-Mtb045 | 315 | 37% | N/A | 214 | 27% | hSNP |
| | BC08-Mtb072 | 379 | 44% | N/A | 188 | 36% | hSNP |
| | BC08-Mtb097 | 329 | 48% | N/A | 267 | 35% | hSNP |
| | BC08-Mtb142 | 260 | 37% | N/A | 482 | 26% | hSNP |
| | BC08-Mtb277 | 221 | 24% | N/A | 308 | 36% | hSNP |
| | BC09-Mtb344 | 264 | 41% | N/A | 177 | 20% | hSNP |
| | BC09-Mtb369 | 244 | 31% | N/A | 157 | 30% | hSNP |
| 2372550 | BC06-Mtb162 | 216 | 22% | hSNP | 210 | 22% | N/A |
| | BC08-Mtb045 | 89 | 0% | REF | 99 | 12% | N/A |
| | BC08-Mtb097 | 151 | 18% | hSNP | 148 | 18% | N/A |
| | BC08-Mtb277 | 180 | 18% | hSNP | 176 | 18% | N/A |
| | BC09-Mtb344 | 143 | 0% | REF | 135 | 0% | N/A |
| | BC09-Mtb369 | 146 | 23% | hSNP | 145 | 22% | N/A |
| | BC10-Mtb020 | 146 | 22% | hSNP | 143 | 22% | N/A |
| | BC10-Mtb053 | 70 | 0% | REF | 67 | 0% | N/A |
| | BC10-Mtb327 | 145 | 0% | REF | 143 | 0% | N/A |
| | BC12-Mtb044 | 175 | 21% | hSNP | 171 | 21% | N/A |
| | BC12-Mtb107 | 134 | 0% | REF | 132 | 0% | N/A |
| | BC12-Mtb274 | 66 | 0% | REF | 63 | 0% | N/A |
| | BC12-Mtb310 | 171 | 23% | hSNP | 164 | 24% | N/A |
| | BC13-Mtb142 | 60 | 0% | REF | 58 | 0% | N/A |
| | BC13-Mtb303 | 136 | 0% | REF | 134 | 0% | N/A |
| | BC13-Mtb451 | 158 | 23% | hSNP | 154 | 23% | N/A |
| | BC14-Mtb181 | 131 | 17% | hSNP | 127 | 17% | N/A |
| | BC14-Mtb263 | 74 | 0% | REF | 84 | 13% | N/A |
| 2401825 | BC04-Mtb018 | 208 | 28% | N/A | 200 | 26% | hSNP |
| | BC05-Mtb035 | 148 | 28% | N/A | 143 | 25% | hSNP |
| | BC06-Mtb081 | 261 | 29% | N/A | 245 | 25% | hSNP |
| | BC06-Mtb162 | 257 | 26% | N/A | 246 | 24% | hSNP |
| | BC07-Mtb028 | 145 | 29% | N/A | 133 | 23% | a |
| | BC08-Mtb045 | 222 | 30% | N/A | 207 | 25% | hSNP |
| | BC08-Mtb097 | 313 | 32% | N/A | 294 | 29% | hSNP |
| | BC08-Mtb277 | 311 | 29% | N/A | 299 | 26% | hSNP |
| | BC09-Mtb344 | 274 | 30% | N/A | 257 | 27% | hSNP |
| | BC09-Mtb369 | 256 | 29% | N/A | 242 | 25% | hSNP |
| | BC10-Mtb020 | 233 | 30% | N/A | 220 | 26% | hSNP |
| | BC10-Mtb053 | 236 | 33% | N/A | 225 | 31% | hSNP |
| | BC12-Mtb044 | 263 | 29% | N/A | 250 | 26% | hSNP |
| | BC12-Mtb274 | 153 | 25% | N/A | 145 | 23% | hSNP |
| | BC12-Mtb310 | 228 | 31% | N/A | 215 | 28% | hSNP |
| | BC13-Mtb142 | 194 | 33% | N/A | 181 | 30% | hSNP |
| | BC13-Mtb303 | 377 | 32% | N/A | 347 | 27% | hSNP |
| | BC13-Mtb451 | 259 | 35% | N/A | 233 | 28% | hSNP |
| | BC14-Mtb181 | 281 | 29% | N/A | 261 | 25% | hSNP |
| | BC14-Mtb263 | 209 | 25% | N/A | 194 | 21% | hSNP |
| 2993651 | BC07-Mtb028 | 100 | 14% | hSNP | 98 | 13% | N/A |
| 4406513 | BC07-Mtb028 | 130 | 14% | N/A | 129 | 14% | hSNP |

Includes loci with a hSNP call in at least one specimen which had a change in at least one variant call following the removal of reads with less than 100 bp
CR: Reads with less than 100 bp aligned to H37Rv.
N/A: Failed filtering protocol in at least one specimen and was removed from analysis.
Ref: Called as reference base, ≤10% of reads support the variant call.
hSNP: Called as a heterogeneous SNP, 10-90% of reads support the variant call.
cSNP: Called as a consensus SNP, ≥90% of reads support the variant call.
INDEL: Called as a small insertion/deletion and therefore was removed from analysis

**Table S4.** Pairwise cSNP distances after final filtering protocol (H37Rv alignment)

| | BC12-Mtb107 | BC08-Mtb142 | BC08-Mtb020 | BC08-Mtb072 | BC10-Mtb327 | BC12-Mtb310 | BC12-Mtb274 | BC13-Mtb303 | BC14-Mtb181 | BC10-Mtb053 | BC14-Mtb263 | BC08-Mtb045 | BC08-Mtb097 | BC08-Mtb277 | BC09-Mtb344 | BC06-Mtb162 | BC13-Mtb451 | BC12-Mtb044 | BC13-Mtb142 | BC09-Mtb369 | BC04-Mtb018 | BC05-Mtb035 | BC10-Mtb020 | BC07-Mtb028 | BC06-Mtb081 |
|---|---|---|---|---|---|---|---|---|---|---|---|---|---|---|---|---|---|---|---|---|---|---|---|---|---|
| **BC12-Mtb107** | 0 | 14 | 14 | 14 | 15 | 1124 | 1118 | 1124 | 1130 | 1133 | 1131 | 1132 | 1137 | 1135 | 1137 | 1143 | 1145 | 1149 | 1151 | 1154 | 1156 | 1156 | 1159 | 1157 | 1158 |
| **BC08-Mtb142** | 14 | 0 | 0 | 0 | 1 | 1126 | 1120 | 1126 | 1132 | 1135 | 1133 | 1134 | 1139 | 1137 | 1139 | 1145 | 1145 | 1151 | 1153 | 1156 | 1158 | 1158 | 1161 | 1159 | 1160 |
| **BC08-Mtb020** | 14 | 0 | 0 | 0 | 1 | 1126 | 1120 | 1126 | 1132 | 1135 | 1133 | 1134 | 1139 | 1137 | 1139 | 1145 | 1145 | 1151 | 1153 | 1156 | 1158 | 1158 | 1161 | 1159 | 1160 |
| **BC08-Mtb072** | 14 | 0 | 0 | 0 | 1 | 1126 | 1120 | 1126 | 1132 | 1135 | 1133 | 1134 | 1139 | 1137 | 1139 | 1145 | 1145 | 1151 | 1153 | 1156 | 1158 | 1158 | 1161 | 1159 | 1160 |
| **BC10-Mtb327** | 15 | 1 | 1 | 1 | 0 | 1127 | 1121 | 1127 | 1133 | 1136 | 1134 | 1135 | 1140 | 1138 | 1140 | 1146 | 1146 | 1152 | 1154 | 1157 | 1159 | 1159 | 1162 | 1160 | 1161 |
| **BC12-Mtb310** | 1124 | 1126 | 1126 | 1126 | 1127 | 0 | 138 | 146 | 190 | 195 | 191 | 192 | 199 | 155 | 197 | 201 | 205 | 209 | 211 | 214 | 216 | 216 | 185 | 217 | 218 |
| **BC12-Mtb274** | 1118 | 1120 | 1120 | 1120 | 1121 | 138 | 0 | 118 | 184 | 189 | 185 | 186 | 193 | 119 | 191 | 197 | 199 | 203 | 205 | 208 | 210 | 210 | 175 | 211 | 212 |
| **BC13-Mtb303** | 1124 | 1126 | 1126 | 1126 | 1127 | 146 | 118 | 0 | 192 | 197 | 193 | 194 | 201 | 135 | 199 | 205 | 207 | 211 | 213 | 216 | 218 | 218 | 185 | 219 | 220 |
| **BC14-Mtb181** | 1130 | 1132 | 1132 | 1132 | 1133 | 190 | 184 | 192 | 0 | 201 | 1 | 196 | 203 | 201 | 201 | 207 | 209 | 213 | 215 | 218 | 218 | 218 | 231 | 219 | 220 |
| **BC10-Mtb053** | 1133 | 1135 | 1135 | 1135 | 1136 | 195 | 189 | 197 | 201 | 0 | 202 | 203 | 210 | 206 | 208 | 214 | 216 | 220 | 222 | 225 | 227 | 227 | 236 | 228 | 229 |
| **BC14-Mtb263** | 1131 | 1133 | 1133 | 1133 | 1134 | 191 | 185 | 193 | 1 | 202 | 0 | 197 | 204 | 202 | 202 | 208 | 210 | 214 | 216 | 219 | 219 | 219 | 232 | 220 | 221 |
| **BC08-mtb045** | 1132 | 1134 | 1134 | 1134 | 1135 | 192 | 186 | 194 | 196 | 203 | 197 | 0 | 113 | 203 | 35 | 117 | 119 | 123 | 125 | 150 | 130 | 130 | 233 | 131 | 132 |
| **BC08-Mtb097** | 1137 | 1139 | 1139 | 1139 | 1140 | 199 | 193 | 201 | 203 | 210 | 204 | 113 | 0 | 210 | 118 | 124 | 126 | 130 | 132 | 157 | 137 | 137 | 240 | 138 | 139 |
| **BC08-Mtb277** | 1135 | 1137 | 1137 | 1137 | 1138 | 155 | 119 | 135 | 201 | 206 | 202 | 203 | 210 | 0 | 208 | 214 | 216 | 218 | 220 | 225 | 225 | 225 | 192 | 226 | 227 |
| **BC09-Mtb344** | 1137 | 1139 | 1139 | 1139 | 1140 | 197 | 191 | 199 | 201 | 208 | 202 | 35 | 118 | 208 | 0 | 122 | 124 | 128 | 130 | 155 | 135 | 135 | 238 | 136 | 137 |
| **BC06-Mtb162** | 1143 | 1145 | 1145 | 1145 | 1146 | 201 | 197 | 205 | 207 | 214 | 208 | 117 | 124 | 214 | 122 | 0 | 130 | 134 | 136 | 161 | 141 | 141 | 244 | 142 | 143 |
| **BC13-Mtb451** | 1145 | 1145 | 1145 | 1145 | 1146 | 205 | 199 | 207 | 209 | 216 | 210 | 119 | 126 | 216 | 124 | 130 | 0 | 118 | 120 | 163 | 143 | 143 | 246 | 144 | 145 |
| **BC12-Mtb044** | 1149 | 1151 | 1151 | 1151 | 1152 | 209 | 203 | 211 | 213 | 220 | 214 | 123 | 130 | 218 | 128 | 134 | 118 | 0 | 2 | 167 | 145 | 145 | 250 | 146 | 147 |
| **BC13-Mtb142** | 1151 | 1153 | 1153 | 1153 | 1154 | 211 | 205 | 213 | 215 | 222 | 216 | 125 | 132 | 220 | 130 | 136 | 120 | 2 | 0 | 169 | 147 | 147 | 252 | 148 | 149 |
| **BC09-Mtb369** | 1154 | 1156 | 1156 | 1156 | 1157 | 214 | 208 | 216 | 218 | 225 | 219 | 150 | 157 | 225 | 155 | 161 | 163 | 167 | 169 | 0 | 174 | 174 | 255 | 175 | 176 |
| **BC04-Mtb018** | 1156 | 1158 | 1158 | 1158 | 1159 | 216 | 210 | 218 | 218 | 227 | 219 | 130 | 137 | 225 | 135 | 141 | 143 | 145 | 147 | 174 | 0 | 0 | 257 | 1 | 2 |
| **BC05-Mtb035** | 1156 | 1158 | 1158 | 1158 | 1159 | 216 | 210 | 218 | 218 | 227 | 219 | 130 | 137 | 225 | 135 | 141 | 143 | 145 | 147 | 174 | 0 | 0 | 257 | 1 | 2 |
| **BC10-Mtb020** | 1159 | 1161 | 1161 | 1161 | 1162 | 185 | 175 | 185 | 231 | 236 | 232 | 233 | 240 | 192 | 238 | 244 | 246 | 250 | 252 | 255 | 257 | 257 | 0 | 258 | 259 |
| **BC07-Mtb028** | 1157 | 1159 | 1159 | 1159 | 1160 | 217 | 211 | 219 | 219 | 228 | 220 | 131 | 138 | 226 | 136 | 142 | 144 | 146 | 148 | 175 | 1 | 1 | 258 | 0 | 1 |
| **BC06-Mtb081** | 1158 | 1160 | 1160 | 1160 | 1161 | 218 | 212 | 220 | 220 | 229 | 221 | 132 | 139 | 227 | 137 | 143 | 145 | 147 | 149 | 176 | 2 | 2 | 259 | 1 | 0 |

**Table S5.** hSNP loci annotations (H37Rv alignment)

| Position | DNA Variant | Protein Variant | Locus Tag | Gene | cSNP Count* | hSNP Count* |
|---|---|---|---|---|---|---|
| 39022 | A>G | N/A | N/A | N/A | 2 | 10 |
| 39030 | C>T | N/A | N/A | N/A | 0 | 20 |
| 57172 | G>A | Asp>Asn | Rv0051 | - | 0 | 1 |
| 67382 | T>C | Leu>Leu | Rv0063 | - | 0 | 1 |
| 336504 | G>T | N/A | N/A | N/A | 20 | 4 |
| 336535 | T>G | N/A | N/A | N/A | 0 | 5 |
| 336537 | T>G | N/A | N/A | N/A | 0 | 5 |
| 336540 | G>T | N/A | N/A | N/A | 0 | 5 |
| 336546 | T>G | N/A | N/A | N/A | 0 | 1 |
| 345757 | A>G | Arg>Arg | Rv0284 | *eccC3* | 0 | 1 |
| 357284 | G>C | Leu>Val | Rv0293c | - | 0 | 1 |
| 752540 | A>T | Ile>Phe | Rv0653c | - | 0 | 1 |
| 778991 | T>C | Val>Ala | Rv0678 | - | 0 | 1 |
| 804332 | C>T | Asp>Asp | Rv0707 | *rpsC* | 0 | 1 |
| 913974 | C>T | Asp>Asn | Rv0821c | *phoY2* | 0 | 1 |
| 996168 | A>C | Val>Gly | Rv0893c | - | 0 | 1 |
| 1171507 | C>T | Arg>His | Rv1048c | - | 0 | 1 |
| 1279337 | C>T | Arg>His | Rv1151c | - | 0 | 1 |
| 1294672 | G>A | Ala>Thr | Rv1165 | *typA* | 0 | 1 |
| 1341083 | C>G | Ala>Ala | Rv1198 | *esxL* | 0 | 1 |
| 1400020 | C>T | Asp>Asp | Rv1253 | *deaD* | 0 | 1 |
| 1407160 | C>A | Val>Phe | Rv1258c | - | 0 | 1 |
| 1480972 | T>C | Glu>Glu | Rv1319c | - | 0 | 25 |
| 1481185 | A>C | Asp>Glu | Rv1319c | - | 0 | 5 |
| 1481337 | G>A | Arg>Trp | Rv1319c | - | 1 | 18 |
| 1569995 | G>T | Arg>Trp | Rv1319c | - | 0 | 1 |
| 1680271 | G>A | Gly>Asp | Rv1490 | - | 0 | 1 |
| 1887029 | T>G | Val>Gly | Rv1663 | *pks17* | 0 | 1 |
| 2076624 | T>C | Leu>Leu | Rv1832 | *gcvB* | 0 | 1 |
| 2133475 | A>G | Val>Ala | Rv1883c | - | 0 | 1 |
| 2196715 | G>C | Val>Leu | Rv1945 | - | 0 | 20 |
| 2196788 | G>A | Gly>Asp | Rv1945 | - | 0 | 4 |
| 2196964 | A>C | Asn>His | Rv1945 | - | 0 | 1 |
| 2245273 | A>G | Lys>Arg | Rv2000 | - | 0 | 1 |
| 2266517 | T>C | Glu>Glu | Rv2020c | - | 0 | 25 |
| 2266550 | G>T | Gly>Gly | Rv2020c | - | 0 | 25 |
| 2266553 | C>G | Ser>Ser | Rv2020c | - | 0 | 25 |
| 2266583 | C>G | Glu>Asp | Rv2020c | - | 0 | 25 |
| 2266598 | G>C | Leu>Leu | Rv2020c | | 0 | 25 |
| 2276945 | C>T | Pro>Pro | Rv2030c | - | 0 | 1 |
| 2310523 | A>T | Leu>Gln | Rv2051c | *ppm1* | 0 | 1 |
| 2360961 | T>C | Phe>Ser | Rv2101 | *helZ* | 0 | 1 |

| | | | | | | |
|---|---|---|---|---|---|---|
| 2401825 | T>C | N/A | N/A | N/A | 0 | 20 |
| 2420904 | G>A | Gly>Gly | Rv2159c | - | 0 | 1 |
| 2432617 | C>T | Thr>Ile | Rv2170 | - | 0 | 1 |
| 2461402 | T>A | N/A | N/A | N/A | 0 | 1 |
| 2491901 | G>A | Thr>Thr | Rv2221c | *glnE* | 1 | 1 |
| 2626018 | T>C | Glu>Gly | Rv2346c | *esx0* | 19 | 1 |
| 2678436 | G>A | Thr>Thr | Rv2385 | *mbtJ* | 0 | 1 |
| 2838806 | C>A | Val>Leu | Rv2522c | - | 0 | 1 |
| 3065692 | T>G | His>Pro | Rv2752c | - | 0 | 1 |
| 3139924 | T>C | Leu>Leu | Rv2833c | *ugpB* | 0 | 1 |
| 3189580 | T>C | N/A | N/A | N/A | 2 | 8 |
| 3191839 | C>G | N/A | N/A | N/A | 0 | 1 |
| 3250262 | C>T | Gly>Gly | Rv2931 | *ppsA* | 0 | 1 |
| 3277391 | G>A | Gly>Gly | Rv2940c | *mas* | 1 | 1 |
| 3375107 | C>A | Leu>Phe | Rv3015c | - | 0 | 1 |
| 3774154 | A>G | Val>Ala | Rv3362c | - | 0 | 1 |
| 3924894 | C>G | Thr>Ser | Rv3506 | *fadD17* | 0 | 1 |
| 4120926 | A>G | Asn>Asp | Rv3680 | - | 0 | 20 |
| 4359135 | T>C | Pro>Pro | Rv3879c | *espK* | 0 | 8 |
| 4395320 | G>A | Gly>Ser | Rv3909 | - | 0 | 1 |
| 4406513 | G>T | Pro>Gln | Rv3918c | *parA* | 0 | 1 |

* Number of specimens the position is variant in out of 25 specimens

**Table S6.** Quality metrics comparing consensus and heterogeneous SNPs observed in lineage 2 specimens

| | **cSNP** | | **hSNP** | |
|---|---|---|---|---|
| | **Mean** | **SD** | **Mean** | **SD** |
| DP | 156.78 | 46.68 | 165.42 | 69.22 |
| QUAL | 227.98 | 0.87 | 214.18 | 22.75 |
| MQ | 59.84 | 1.19 | 59.32 | 0.98 |
| SP | 0.13 | 0.93 | 10.12 | 9.50 |
| BQB* | 0.98 | 0.11 | 0.64 | 0.32 |
| MQB* | 1.00 | 0.02 | 0.85 | 0.20 |
| RPB* | 0.98 | 0.10 | 0.24 | 0.10 |

DP: High quality read depth

QUAL: Recalibrated base quality score

MQ: Average mapping quality

SP: Phred-scaled strand bias P-value

BQB: Mann-Whitney U test of Base Quality Bias

MQB: Mann-Whitney U test of Mapping Quality Bias

RPB: Mann-Whitney U test of Read Position Bias

*As BQB, MQB, and RPB are only defined at positions with reference and variant reads, assumes an RPB value of 1.0 for SNPs with 100% variant reads

**Table S7.** hSNP loci annotations (CCDC5079 alignment)

| Position | DNA Variant | Protein Variant | Locus Tag | cSNP Count* | hSNP Count* |
|---|---|---|---|---|---|
| 58205 | G>A | Asp>Asn | 48 | 0 | 1 |
| 68416 | T>C | Leu>Leu | 0056 | 0 | 1 |
| 101511 | C>G | Leu>Leu | 0083 | 0 | 1 |
| 101514 | C>G | Glu>Asp | 0083 | 0 | 1 |
| 334181 | C>A | N/A | N/A | 0 | 1 |
| 343409 | A>G | Arg>Arg | 0264 | 0 | 1 |
| 354934 | G>C | Leu>Val | 0272 | 0 | 1 |
| 750110 | A>T | Ile>Phe | 0613 | 0 | 1 |
| 776562 | T>C | Val>Ala | 0630 | 0 | 1 |
| 801957 | C>T | Asp>Asp | 0656 | 0 | 1 |
| 910189 | C>T | Val>Gly | 0825 | 0 | 1 |
| 961162 | G>C | Leu>Val | 0802 | 9 | 7 |
| 992401 | A>C | Val>Gly | 0825 | 0 | 1 |
| 1167736 | C>T | Arg>His | 0969 | 0 | 1 |
| 1276930 | C>T | Arg>His | 1063 | 0 | 1 |
| 1292267 | G>A | Ala>Thr | 1078 | 0 | 1 |
| 1338176 | T>C | N/A | N/A | 0 | 2 |
| 1397618 | C>T | Asp>Asp | 1156 | 0 | 1 |
| 1636214 | G>C | Ala>Gly | 1349 | 0 | 3 |
| 1679556 | G>A | Gly>Asp | 1386 | 0 | 1 |
| 1805016 | G>A | N/A | N/A | 0 | 20 |
| 1877091 | T>G | Val>Gly | 1542 | 0 | 1 |
| 2058197 | T>C | Lue>Leu | 1689 | 0 | 1 |
| 2232180 | A>G | N/A | N/A | 0 | 1 |
| 2270208 | C>T | Pro>Pro | 1878 | 0 | 1 |
| 2288942 | A>C | Leu>Val | 1896 | 4 | 14 |
| 2288949 | C>T | Val>Val | 1896 | 0 | 16 |
| 2289299 | G>C | Pro>Ala | 1896 | 0 | 20 |
| 2289438 | A>G | Gly>Gly | 1896 | 0 | 20 |
| 2290131 | G>A | Gly>Gly | 1896 | 0 | 20 |
| 2290135 | C>T | Arg>His | 1896 | 0 | 20 |
| 2290140 | G>C | Arg>Arg | 1896 | 0 | 20 |
| 2290364 | G>A | Leu>Leu | 1896 | 1 | 19 |
| 2290365 | C>A | ValVval | 1896 | 1 | 19 |
| 2291184 | C>A | Val>Val | 1896 | 0 | 20 |
| 2291190 | C>A | Val>Val | 1896 | 0 | 20 |
| 2291193 | C>A | Ser>Ser | 1896 | 0 | 20 |
| 2291196 | A>G | Gly>Gly | 1896 | 0 | 20 |
| 2291201 | A>T | Ser>Thr | 1896 | 0 | 20 |
| 2291202 | T>G | Ala>Ala | 1896 | 0 | 20 |
| 2291213 | C>G | Val>Leu | 1896 | 0 | 20 |
| 2291216 | A>G | Leu>Leu | 1896 | 0 | 20 |
| 2291217 | C>G | Pro>Pro | 1896 | 0 | 20 |

| | | | | | |
|---|---|---|---|---|---|
| 2291220 | CT | Ala>Ala | 1896 | 0 | 20 |
| 2291232 | T>C | Leu>Leu | 1896 | 0 | 20 |
| 2291234 | A>G | Leu>Leu | 1896 | 0 | 20 |
| 2291235 | T>C | Glu>Glu | 1896 | 0 | 20 |
| 2291282 | G>A | Leu>Leu | 1896 | 0 | 20 |
| 2291289 | G>A | Phe.Phe | 1896 | 0 | 20 |
| 2291295 | C>G | Ala>Ala | 1896 | 0 | 20 |
| 2291297 | C>T | Ala>Thr | 1896 | 0 | 20 |
| 2291298 | T>G | Gly>Gly | 1896 | 0 | 20 |
| 2291307 | C>G | Leu>Leu | 1896 | 0 | 20 |
| 2291309 | G>T | Leu>Met | 1896 | 0 | 20 |
| 2291311 | A>G | Val>Vala | 1896 | 0 | 20 |
| 2291312 | C>T | Val>Met | 1896 | 0 | 20 |
| 2291317 | T>C | Asp>Gly | 1896 | 0 | 20 |
| 2291319 | T>G | Ser>Ser | 1896 | 0 | 20 |
| 2291321 | A>C | Ser>Ala | 1896 | 0 | 20 |
| 2291327 | A>C | Ser>Ala | 1896 | 0 | 20 |
| 2291343 | C>G | Pro>Pro | 1896 | 0 | 20 |
| 2291344 | G>C | Pro>Arg | 1896 | 0 | 20 |
| 2291345 | G>C | Pro>Ala | 1896 | 0 | 20 |
| 2291353 | T>C | Asn>Ser | 1896 | 0 | 20 |
| 2291363 | A>G | Leu>Leu | 1896 | 0 | 20 |
| 2293521 | A>G | Ala>Ala | 1896 | 0 | 20 |
| 2297739 | A>T | Leu>Gln | 1899 | 0 | 1 |
| 2334635 | C>T | Pro>Leu | 1932 | 0 | 20 |
| 2348077 | T>C | Phe>Ser | 1943 | 0 | 1 |
| 2409609 | G>A | Gly>Gly | 1997 | 0 | 1 |
| 2479141 | G>A | Thr>Thr | 2058 | 1 | 1 |
| 2661801 | G>A | Thr>Thr | 2205 | 0 | 1 |
| 2820606 | C>A | Val>Leu | 2324 | 0 | 1 |
| 3046387 | T>G | His>Pro | 2526 | 0 | 1 |
| 3114366 | T>C | Leu>Leu | 2594 | 0 | 1 |
| 3164025 | T>C | N/A | N/A | 2 | 8 |
| 3166283 | C>G | N/A | N/A | 0 | 1 |
| 3225204 | C>T | Gly>Gly | 2690 | 0 | 1 |
| 3252334 | G>A | Gly>Gly | 2700 | 1 | 1 |
| 3350171 | C>A | Leu>Phe | 2770 | 0 | 1 |
| 3733144 | T>G | Asn>Thr | 3096 | 0 | 1 |
| 3737137 | T>G | N/A | N/A | 19 | 1 |
| 3758127 | A>G | Val>Ala | 3110 | 0 | 1 |
| 3911593 | C>G | Thr>Ser | 3242 | 0 | 1 |
| 4346412 | C>T | Arg>Trp | 3607 | 0 | 1 |
| 4382601 | G>A | Gly>Ser | 3636 | 0 | 1 |
| 4393793 | G>T | Pro>Gln | 3645 | 0 | 1 |

* Number of specimens the position is variant in out of 25 specimens

**Table S8.** Informative SNP loci annotations in serial specimens from Patient A (H37Rv alignment)

| Position | DNA Variant | Protein Variant | Locus Tag | Gene | Variant read frequency (variant reads/total reads) 2004 | 2005 | 2006 | 2007 |
|---|---|---|---|---|---|---|---|---|
| 155388 | C>T | Ala>Val | Rv0127 | *mak* | 0% (0/161) | 0% (0/166) | 93% (142/152) | 91% (92/97) |
| 357284 | G>C | Leu>Val | Rv0293c | - | 0% (0/191) | 0% (0/158) | 0% (0/165) | 20% (22/106) |
| 778991 | T>C | Val>Ala | Rv0678 | - | 0% (0/157) | 0% (0/161) | 0% (0/180) | 26% (28/108) |
| 1171507 | C>T | Arg>His | Rv1048c | - | 38% (57/149) | 0% (0/127) | 0% (0/158) | 0% (0/100) |
| 1279337 | C>T | Arg>His | Rv1151c | - | 0% (0/162) | 0% (0/195) | 0% (0/164) | 16% (15/91) |
| 1294672 | G>A | Ala>Thr | Rv1165 | *typA* | 0% (0/157) | 0% (0/126) | 0% (0/172) | 14% (14/100) |
| 1400020 | C>T | Asp>Asp | Rv1253 | *deaD* | 0% (0/174) | 0% (0/125) | 0% (0/167) | 22% (16/73) |
| 2245273 | A>G | Lys>Arg | Rv2000 | - | 0% (0/143) | 0% (0/112) | 0% (0/173) | 20% (16/79) |
| 2310523 | A>T | Leu>Gln | Rv2051c | *ppm1* | 0% (0/165) | 0% (0/155) | 0% (0/147) | 19% (19/101) |
| 3277391 | G>A | Gly>Gly | Rv2940c | *mas* | 0% (0/157) | 0% (0/196) | 91% (145/159) | 82% (83/101) |
| 3924894 | C>G | Thr>Ser | Rv3506 | *fadD17* | 0% (0/180) | 0% (0/109) | 0% (0/155) | 18% (18/101) |
| 4406513 | G>T | Pro>Gln | Rv3918c | *parA* | 0% (0/174) | 0% (0/146) | 0% (0/191) | 14% (18/129) |
